# A Validated Set of Fluorescent-Protein-Based Markers for Major Organelles in Yeast (Saccharomyces cerevisiae)

**DOI:** 10.1128/mBio.01691-19

**Published:** 2019-09-03

**Authors:** Jing Zhu, Zheng-Tan Zhang, Shi-Wei Tang, Bo-Song Zhao, Hui Li, Jing-Zhen Song, Dan Li, Zhiping Xie

**Affiliations:** a State Key Laboratory of Microbial Metabolism & Joint International Research Laboratory of Metabolic & Developmental Sciences, School of Life Sciences and Biotechnology, Shanghai Jiao Tong University, Shanghai, People’s Republic of China; University of Chicago; Cornell University

**Keywords:** organelle, fluorescent protein, nomenclature, yeast, validation

## Abstract

Eukaryotic cells share a basic scheme of internal organization featuring membrane-based organelles. The use of fluorescent proteins (FPs) greatly facilitated live-cell imaging of organelle dynamics and protein trafficking. One major limitation of this approach is that the fusion of an FP to a target protein can and often does compromise the function of the target protein and alter its subcellular localization. The optimization process to obtain a desirable fusion construct can be time-consuming or even unsuccessful. In this work, we set out to provide a validated set of FP-based markers for major organelles in the budding yeast (Saccharomyces cerevisiae). Out of over 160 plasmids constructed, we present a final set of 42 plasmids, the recommendations for which are backed up by meticulous evaluations. The tool set includes three colors (green, red, and blue) and covers the endoplasmic reticulum (ER), nucleus, Golgi apparatus, endosomes, vacuoles, mitochondria, peroxisomes, and lipid droplets. The fidelity of the markers was established by systematic cross-comparison and quantification. Functional assays were performed to examine the impact of marker expression on the secretory pathway, endocytic pathway, and metabolic activities of mitochondria and peroxisomes. Concomitantly, our work constitutes a reassessment of organelle identities in this model organism. Our data support the recognition that “late Golgi” and “early endosomes,” two seemingly distinct terms, denote the same compartment in yeast. Conversely, all other organelles can be visually separated from each other at the resolution of conventional light microscopy, and quantification results justify their classification as distinct entities.

## INTRODUCTION

Subcellular compartmentalization into membrane-based organelles is a hallmark of eukaryotic cells (1, 2). The budding yeast Saccharomyces cerevisiae is a widely used model organism in the study of organelle functions and intracellular trafficking pathways. Many important discoveries, including the genetic characterization of the secretory pathway, peroxisome biogenesis, and autophagy, originated from yeast studies (3–5). Even for such a relatively simple organism, findings that challenge existing paradigms of the endomembrane system continue to emerge. For instance, the peroxisomes were long thought to form autonomously by growth and division of preexisting units. However, growing evidence now favors a model in which vesicular trafficking from the endoplasmic reticulum serves as a major pathway of peroxisomal membrane protein biogenesis (6). Similarly, “early endosomes” and “late Golgi” were originally considered to be distinct organelles, one being the destination of endocytic vesicles and the other being the last stopover before secretion. The concept was partly based on generalization from animal studies. However, Day et al. recently argued that the two are actually the same entity in yeast (7). Such cases reflect the insufficiency in our knowledge regarding the internal organization of cells and call for more thorough endeavors in future investigations.

Organelle markers, especially those applicable in live cells, are among the most basic tools in cell biology. They are often employed to track the dynamics of the corresponding organelles or used to pinpoint the subcellular localization of a given macromolecule. The rapid development of fluorescent proteins has propelled their widespread use as fusion partners in live-cell imaging (8). Since the 1990s, many variants of fluorescent proteins have been developed, each with distinct advantages. Convenient as it is, a potential risk is that fluorescent protein tagging may interfere with intracellular trafficking of the target protein, in particular when using variants other than green fluorescent protein (GFP) (9–11). There is yet to be a solid trend or a design principle where the use of a particular variant is always better than another in terms of the resulting protein functionality and targeting specificity. As a result, obtaining a suitable labeling construct often becomes an odyssey of trial and error.

In the present study, we aim to provide a validated set of fluorescent-protein-based markers for major organelles in budding yeast. We hope by performing thorough quantification and functional evaluations, our work will save some time for colleagues in this field. Furthermore, our work constitutes a comprehensive evaluation of organelle identities.

## RESULTS

### Overview.

The internal organization of a yeast cell represents a simplified version of typical eukaryotic cells. Its major membrane-bound organelles include the nucleus, endoplasmic reticulum (ER), Golgi apparatus, endosomes, vacuoles, mitochondria, peroxisomes, and lipid droplets. During the course of this work, we constructed over 160 fluorescent protein chimeras. Our primary criterion for the evaluation of their performance is the labeling fidelity, which was deduced from systematic cross-comparison with other chimeras. We also took into consideration the brightness and functionality of the constructs, the latter of which was inferred from protein trafficking assays, organelle function assessments, and morphological observations. The result is a collection of reasonably well-performing organelle green and red markers and a set of somewhat-usable blue markers (Table 1). Unless otherwise noted, the chimeras are expressed under the control of their endogenous promoters.

**TABLE 1:** Validated markers for major yeast organelles

Organelle	Marker for color:		
	Green	Red	Blue
Endoplasmic reticulum	Emc1-2GFP	Elo3-mCherry	Sec63-2mTagBFP2
	SS-GFP-HDEL^*a*^	SS-mCherry-HDEL^*a*^	Elo3-mTagBFP2
Nucleus	Nab2-GFP	Nab2-mCherry	Nab2-mTagBFP2
Early Golgi	GFP-Sed5	Anp1-mCherry	Mnn9-mTagBFP2
	Vrg4-GFP		Sec26-mTagBFP2
	Anp1-GFP		
Late Golgi/early endosome	Chs5-GFP	Sec7-DuDre	Sec7-mTagBFP2
	Sec7-2GFP^*b*^	Chs5-mCherry	mTagBFP2-Tlg1
	GFP-Tlg1^*a*^		
	GFP-Tlg2^*a*^		
Late endosome	Vps4-GFP	Vps4-DuDre	Vps4-mTagBFP2
	GFP-Pep12	Snf7-mCherry	
Vacuole	GFP-Pho8^*a*^	Vph1-mCherry	Vph1-mTagBFP2
	Vph1-2GFP^*b*^		
Mitochondria	Cox4-GFP	Cox4-DuDre	Cox4-mTagBFP2
			Cox9-mTagBFP2
Peroxisome	Pex1-2GFP	Pex3-DuDre	mTagBFP2-SKL^*a*^
Lipid droplet	Tgl3-GFP	Tgl3-mCherry	
		Erg6-mCherry	

a Except for those marked, all other constructs are expressed under the control of respective endogenous promoters.

b These two constructs are designed to insert fluorescent proteins at the C termini of endogenous ORFs. All of the other constructs are designed to integrate as an additional copy in the genome, with the endogenous counterparts still present.

For clarity purposes, we begin by describing the green and red markers and then move on to the blue markers.

### ER and nucleus.

**FIG 1:**
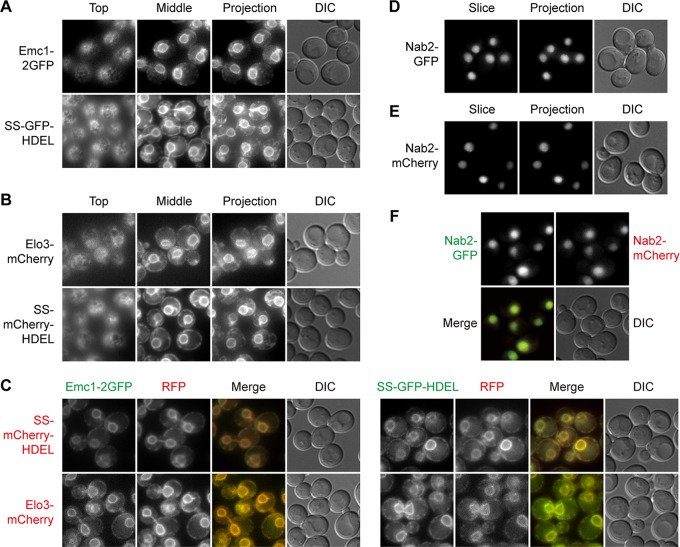
Green and red markers of endoplasmic reticulum and nucleus. Mid-log-phase yeast cells expressing the indicated fluorescent protein fusion constructs were immobilized on concanavalin A-coated cover glass. z-stacks containing 15 slices at 0.5-μm step size were captured. Top, slice showing the top of the cells; middle, slice going through the center of cells; slice, a single representative slice; projection, maximal intensity projection of z-stacks; DIC, differential interference contrast. For colocalization experiments, images shown are slices. (A and B) Green (A) and red (B) markers of the ER. The tubular structures of the cortical ER are visible in the top slices. (C) Colocalization between green and red ER markers. (D and E) Green (D) and red (E) markers of the nucleus. (F) Colocalization between green and red nucleus markers.

The ER is the starting point of the secretory pathway. Most secreted proteins and resident proteins of the Golgi apparatus, endosomes, and vacuoles begin their journey in the endomembrane system from the ER. Under a light microscope, the ER structures in yeast cells can be categorized into the following three populations: nuclear ER that continues from the nuclear envelope, cortical ER underneath the plasma membrane, and intermediate structures connecting the two (12). Emc1 is a member of the conserved ER-membrane protein complex (EMC) (13). Elo3 is a fatty acid enlongase (14). Both Emc1-2GFP and Elo3-mCherry labeled all three populations of the ER, and they colocalized with each other (Fig. 1A to C) (15). In addition, we made signal sequence (SS)-GFP/mCherry-HDEL constructs driven by the strong *TPI1* promoter (Fig. 1A to C) (16), whose brighter signal is advantageous in time-lapse imaging.

We also constructed chimeras of Nab2 for labeling of the nucleus. Nab2 is a nuclear polyadenylated RNA-binding protein (17). Live-cell imaging indicated that Nab2-GFP and Nab2-mCherry colocalized with each other (Fig. 1D to F) and labeled the same 4′,6-diamidino-phenylindole (DAPI)-positive compartment (data not shown).

### Early Golgi.

**FIG 2:**
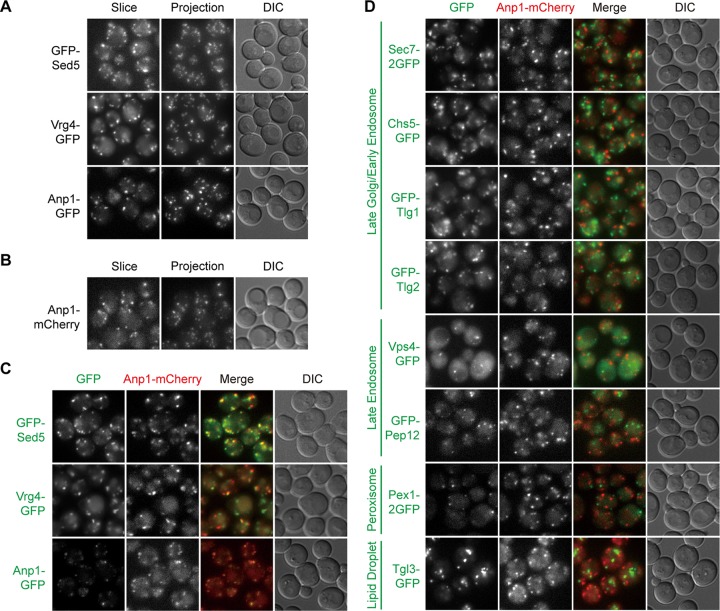
Green and red markers of early Golgi. Images were captured and presented as in Fig. 1. (A and B) Green (A) and red (B) markers of the early Golgi. (C) Colocalization between green and red early Golgi markers. (D) Lack of colocalization between green markers of other organelles and the red marker of early Golgi.

The Golgi apparatus serves as the second stop of trafficking for most proteins *en route* to the endosomes, vacuoles, and plasma membrane. In yeast cells, the Golgi cisternae are scattered in the cytoplasm without the typical stack structure seen in many other species. The early Golgi receives cargos from the ER, whereas the late Golgi sorts cargos into specific outgoing vesicles. The physical separation of the cisternae enabled researchers to visualize the maturation process from the early Golgi to the late Golgi without the need for superresolution techniques (18, 19). Among candidate constructs, we found that GFP-Sed5, Vrg4-GFP, and Anp1-GFP displayed numerous punctate signals (Fig. 2A). Sed5 is a target-SNAP receptor (t-SNARE) of the early Golgi (20–22). Vrg4 is a Golgi GDP-mannose transporter (23). Anp1 is a subunit of the alpha-1,6 mannosyltransferase complex (24). Of the three, the signal of Vrg4-GFP was the strongest, albeit with some additional vacuolar accumulation. The signal of Anp1-GFP was the weakest. All three colocalized well with Anp1-mCherry (Fig. 2B and C), indicating that these four constructs label the same compartment. In contrast, little colocalization was observed between Anp1-mCherry and GFP fusion proteins labeling the late Golgi, late endosomes, peroxisomes, and lipid droplets, even though all of them were also visualized as multiple puncta in the cytoplasm (Fig. 2D). These data are consistent with the early Golgi being a distinct entity in yeast cells.

### Late Golgi/early endosomes.

**FIG 3:**
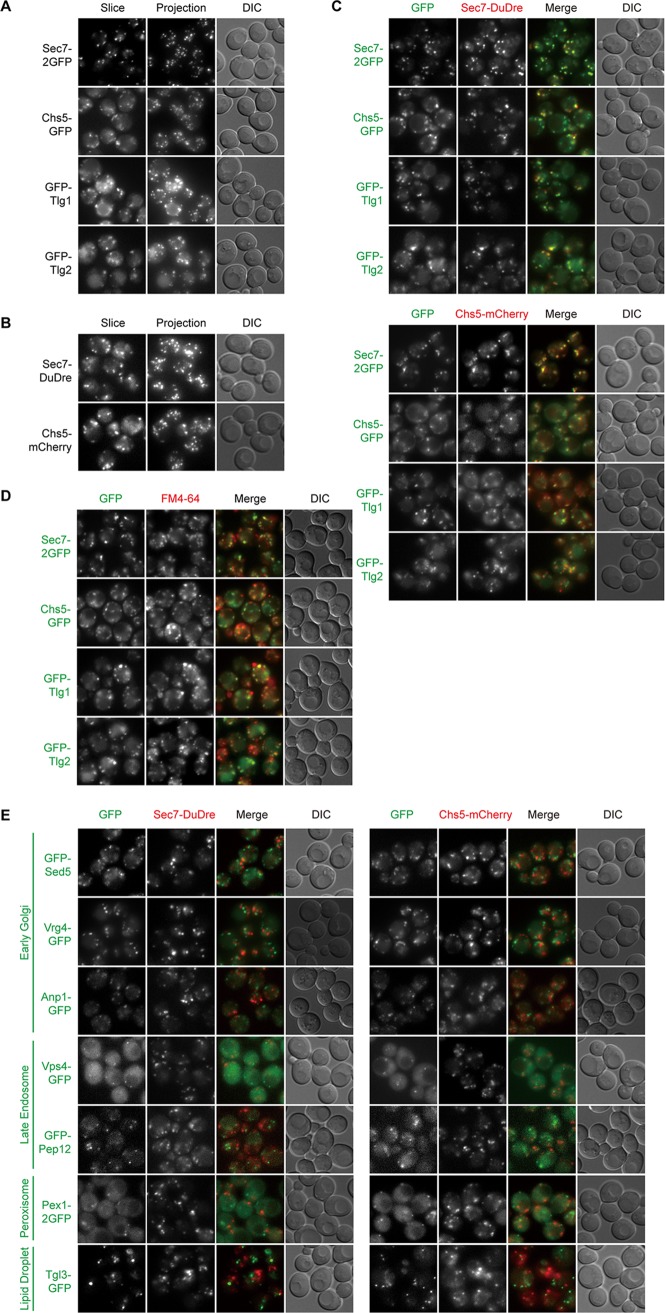
Green and red markers of late Golgi/early endosomes. Images were captured and presented as in Fig. 1. (A and B) Green (A) and red (B) markers of late Golgi/early endosomes. (C) Colocalization between green and red late Golgi/early endosome markers. (D) Colocalization between green late Golgi/early endosomes markers and FM4-64 puncta. (E) Lack of colocalization between green markers of other organelles and red markers of late Golgi/early endosomes.

Among candidate constructs of the late Golgi, we found that Sec7-2GFP, Chs5-GFP, Sec7-DuDre, and Chs5-mCherry all produced good signals (Fig. 3A and B) (DuDre is a tandem construct of DsRed Express 2) (25, 26). Sec7 is a guanine nucleotide exchange factor (GEF) for ADP ribosylation factors (ARF) (27). Chs5 is a subunit of the exomer complex (28, 29). Cross-comparison demonstrated that all four constructs labeled the same compartment (Fig. 3C). In contrast, for Sec7-DuDre and Chs5-mCherry, little colocalization with green markers of the early Golgi, late endosomes, peroxisomes, and lipid droplets was detected (Fig. 3E).

A notable exception in this case was that red constructs of Sec7 and Chs5 displayed efficient colocalization with green constructs of Tlg1 and Tlg2, two t-SNARE proteins supposedly present on early endosomes (Fig. 3C) (30, 31). We then used FM4-64 to label compartments of endocytic origin and found that a substantial percentage of FM4-64-positive structures contained the green Tlg chimeras (mean ± standard deviation, 64% ± 5% with Tlg1 and 55% ± 2% with Tlg2; *n* = 3) (Fig. 3D), confirming that our constructs behave similarly to those in the literature. Furthermore, we noticed that Sec7 and Chs5 puncta were also FM4-64 positive (83% ± 5% for Sec7 and 58% ± 6% for Chs5, using FM4-64 dot numbers as the denominators). Based on our cross-comparison results (Fig. 3C and E), we are reasonably confident that Sec7 and Chs5 label the late Golgi. Therefore, we interpret the colocalization of Tlg constructs with Sec7 and Chs5 as indication that “late Golgi” and “early endosomes” described in the existing yeast literature actually refer to the same compartment. These data support the recent hypothesis presented by Day et al. (7).

### Late endosomes.

**FIG 4:**
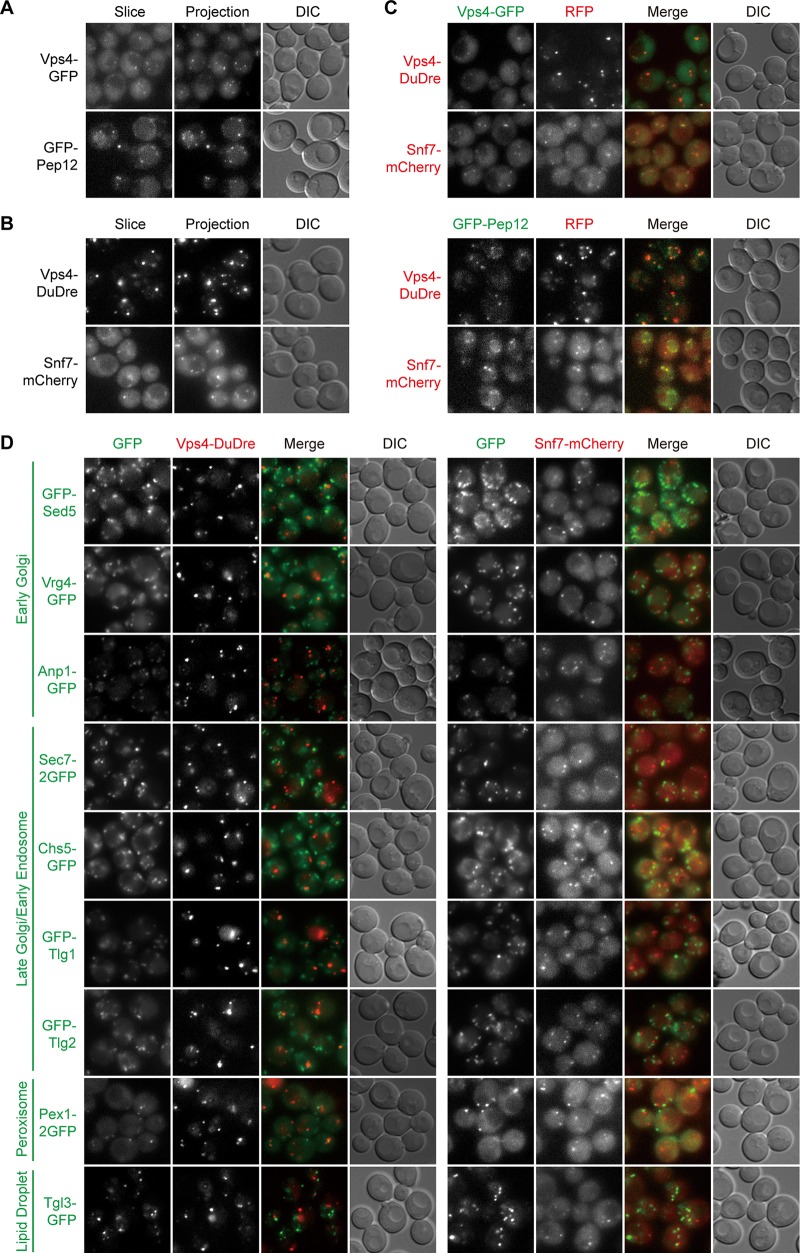
Green and red markers of late endosomes. Images were captured and presented as in Fig. 1. (A and B) Green (A) and red (B) markers of late endosomes. (C) Colocalization between green and red late endosome markers. (D) Lack of colocalization between green markers of other organelles and the red markers of late endosomes.

The late endosomes, also known as the multivesicular bodies (MVB) and the prevacuolar compartments (PVC), are a key hub of protein sorting. Their residents include the retromer complex and the endosomal sorting complexes required for transport (ESCRT) complexes, which mediate cargo recycling and internalization, respectively (7, 32, 33). Cross-comparison results indicated that Vps4-GFP, GFP-Pep12, Vps4-DuDre, and Snf7-mCherry labeled late endosomes (Fig. 4A to C). Vps4 is an AAA-ATPase that catalyzes ESCRT-III disassembly (34). Pep12 is a t-SNARE on late endosomes (35, 36). Snf7 is a subunit of the ESCRT-III complex (37). In addition to punctate signals, some GFP-Pep12 was present on the vacuole (Fig. 4A). Nevertheless, with regard to punctate signals, we observed good colocalization between the green constructs of Vps4 and Pep12 and the red constructs of Vps4 and Snf7. Little colocalization was present between green markers of the early Golgi, the late Golgi/early endosomes, peroxisomes, and lipid droplets, and the two red constructs of Vps4 and Snf7 (Fig. 4D).

For these late endosome markers, constructs containing a single copy of fluorescent protein produced weak signals. The fusion of Vps4 to DuDre, a tandem construct of DsRed Express 2 (25, 26), not only improved signal intensity but also appeared to augment the membrane recruitment of Vps4, resulting in diminished cytosolic distribution (Fig. 4A and B). In the case of Snf7, we found that its GFP chimera labeled both late endosomes and vacuoles (see Table S1 in the supplemental material), whereas its mCherry chimera primarily resided on late endosomes. We therefore advise caution when using these two red constructs (see also “Function analysis,” below, regarding Snf7-mCherry).

10.1128/mBio.01691-19.9 TABLE S1 Fusion constructs with suboptimal properties. This table contains brief explanations of why each of the listed constructs is unsuitable as an organelle marker under our experimental conditions. Download Table S1, DOCX file, 0.1 MB. Copyright © 2019 Zhu et al. 2019 Zhu et al. This content is distributed under the terms of the Creative Commons Attribution 4.0 International license.

### Vacuoles.

**FIG 5:**
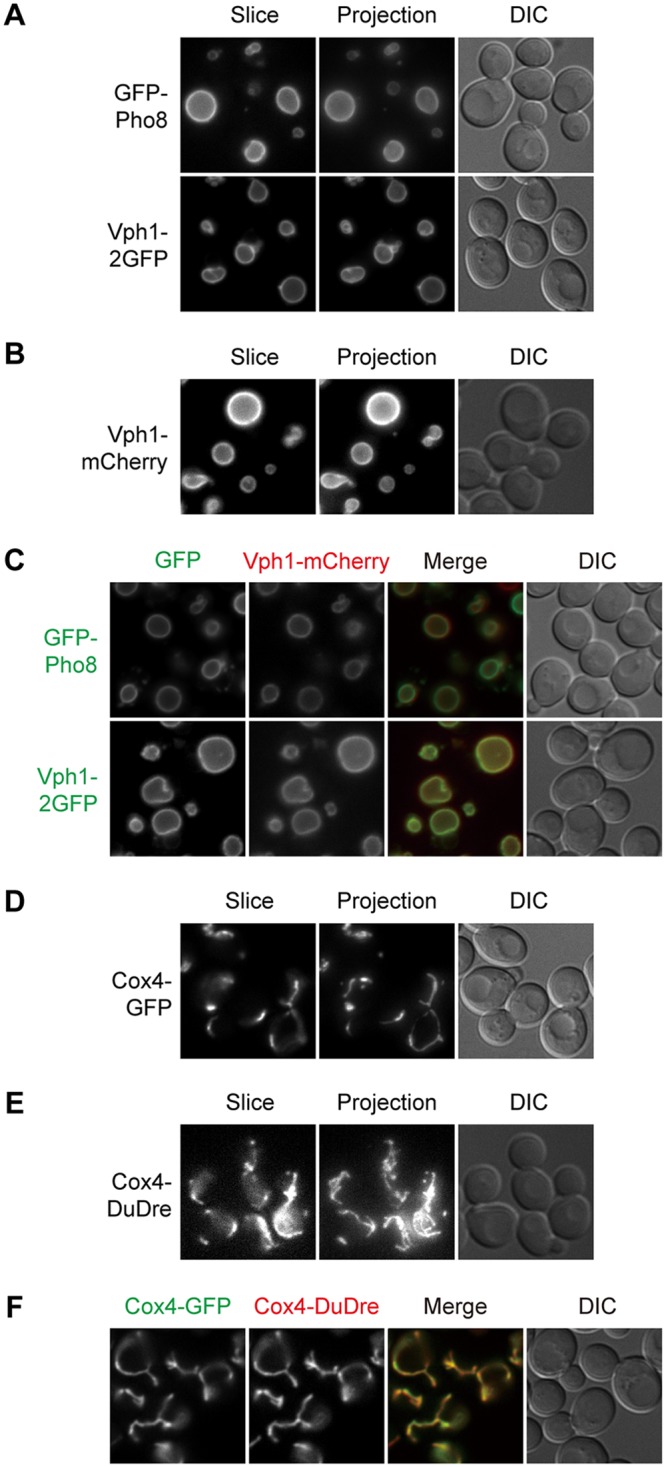
Green and red markers of vacuoles and mitochondria. Images were captured and presented as in Fig. 1. (A and B) Green (A) and red (B) markers of vacuoles. (C) Colocalization between green and red vacuole markers. (D and E) Green (D) and red (E) markers of mitochondria. (F) Colocalization between green and red mitochondrial markers.

Vacuoles in yeast are functional analogues of lysosomes in animals. They undergo frequent fission and fusion, the balance of which determines the steady-state morphology of vacuoles. Under our experimental conditions, most cells contained a single large vacuole visible under differential interference contrast (DIC) imaging. Two routes deliver proteins to the vacuolar membrane, one through late endosomes, and the other directly from the late Golgi (38). We picked Vph1 and Pho8 as representative cargos of the two routes (39, 40). Vph1 is a subunit of the vacuolar proton pump (41). Pho8 is a vacuolar alkaline phosphatase (42). Vph1-2GFP, GFP-Pho8, and Vph1-mCherry all labeled the vacuolar limiting membrane efficiently and displayed nearly perfect colocalization between the green and red constructs (Fig. 5A to C).

### Mitochondria.

Like the ER and vacuoles, mitochondria are another example of organelles with distinct morphologies, making it easier to judge labeling fidelity. Cox4 is a subunit of cytochrome *c* oxidase (43). Signals of Cox4-GFP and Cox4-DuDre possessed the typical thread-like contour of mitochondria and colocalized with each other (Fig. 5D to F). Structures containing Cox4-GFP also stained positive for MitoTracker red (data not shown).

### Peroxisomes and lipid droplets.

**FIG 6:**
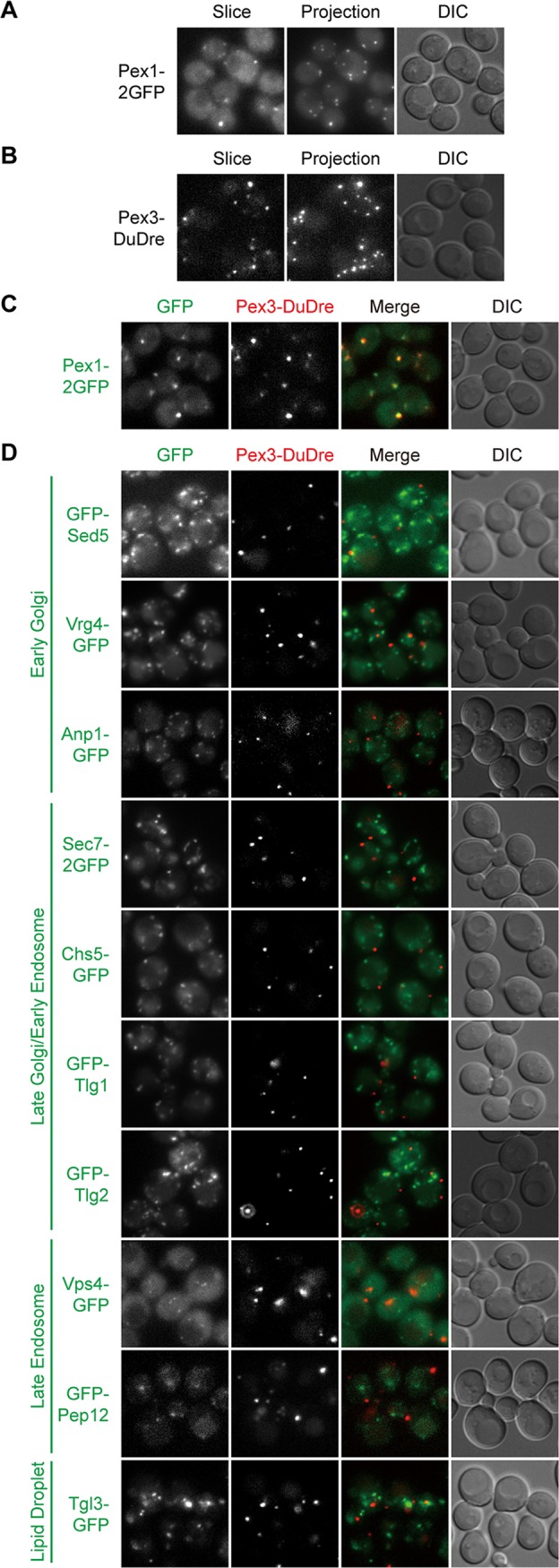
Green and red markers of peroxisomes. Images were captured and presented as in Fig. 1. (A and B) Green (A) and red (B) markers of peroxisomes. (C) Colocalization between green and red peroxisome markers. (D) Lack of colocalization between green markers of other organelles and the red marker of peroxisomes.

**FIG 7:**
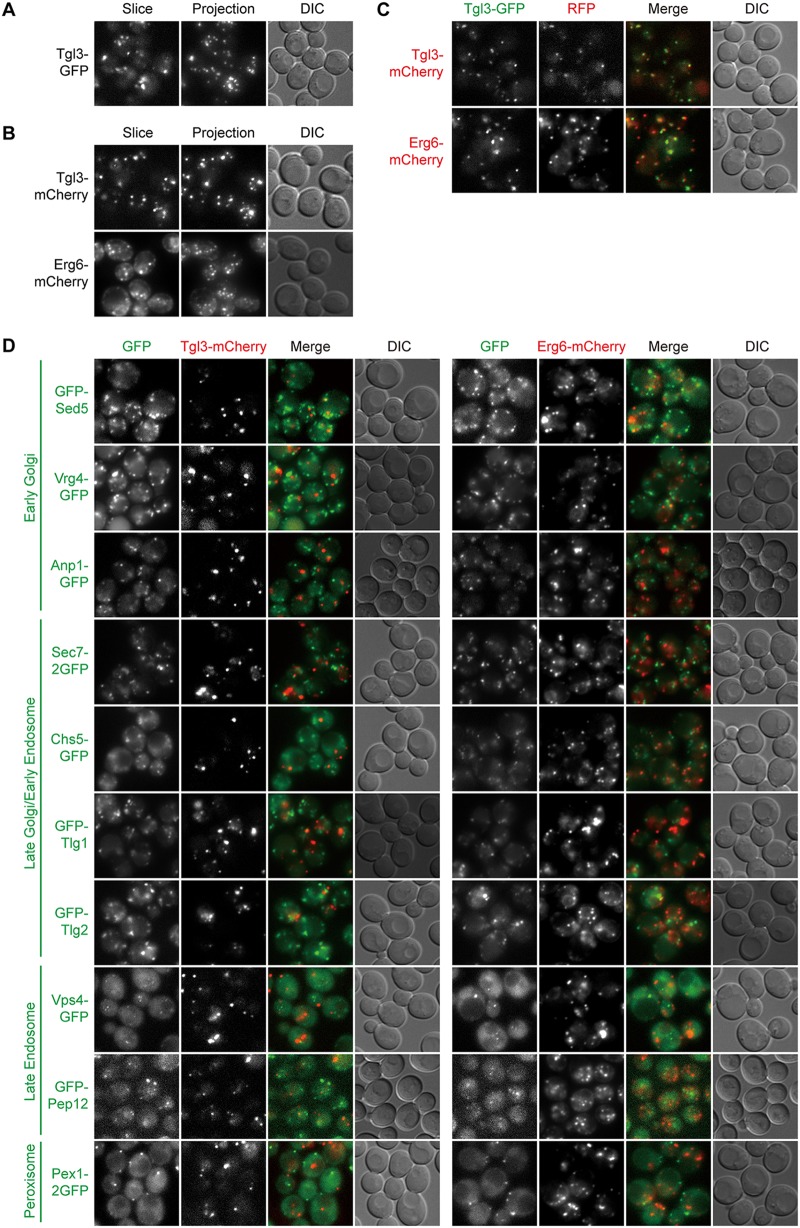
Green and red markers of lipid droplets. Images were captured and presented as in Fig. 1. (A and B) Green (A) and red (B) markers of lipid droplets. (C) Colocalization between green and red lipid droplets markers. (D) Lack of colocalization between green markers of other organelles and the red markers of lipid droplets.

Both peroxisomes and lipid droplets are organelles with key roles in lipid metabolism. Their biogenesis is intricately associated with the ER, although the precise mechanisms are still under intense investigation (6, 44). After testing several candidates, we settled on Pex1-2GFP and Pex3-DuDre as markers of peroxisomes and Tgl3-GFP, Tgl3-mCherry, and Erg6-mCherry as markers of lipid droplets. Pex1 is an AAA-ATPase involved in the recycling of peroxisomal matrix protein receptor (45). Pex3 is a peroxisomal membrane protein functioning in the proper targeting of peroxisomal membrane proteins (45). Tgl3 is a triacylglycerol lipase (46). Erg6 is an enzyme in the ergosterol biosynthesis pathway (46). Pex3-DuDre, Tgl3-mCherry, and Erg6-mCherry colocalized with green constructs of the intended organelles but not markers of other organelles with punctate distributions (Fig. 6A to D and 7A to D). Both Tgl3-mCherry and Erg6-mCherry also displayed good colocalization with the BODIPY signal (data not shown).

### Quantification of colocalization.

**FIG 8:**
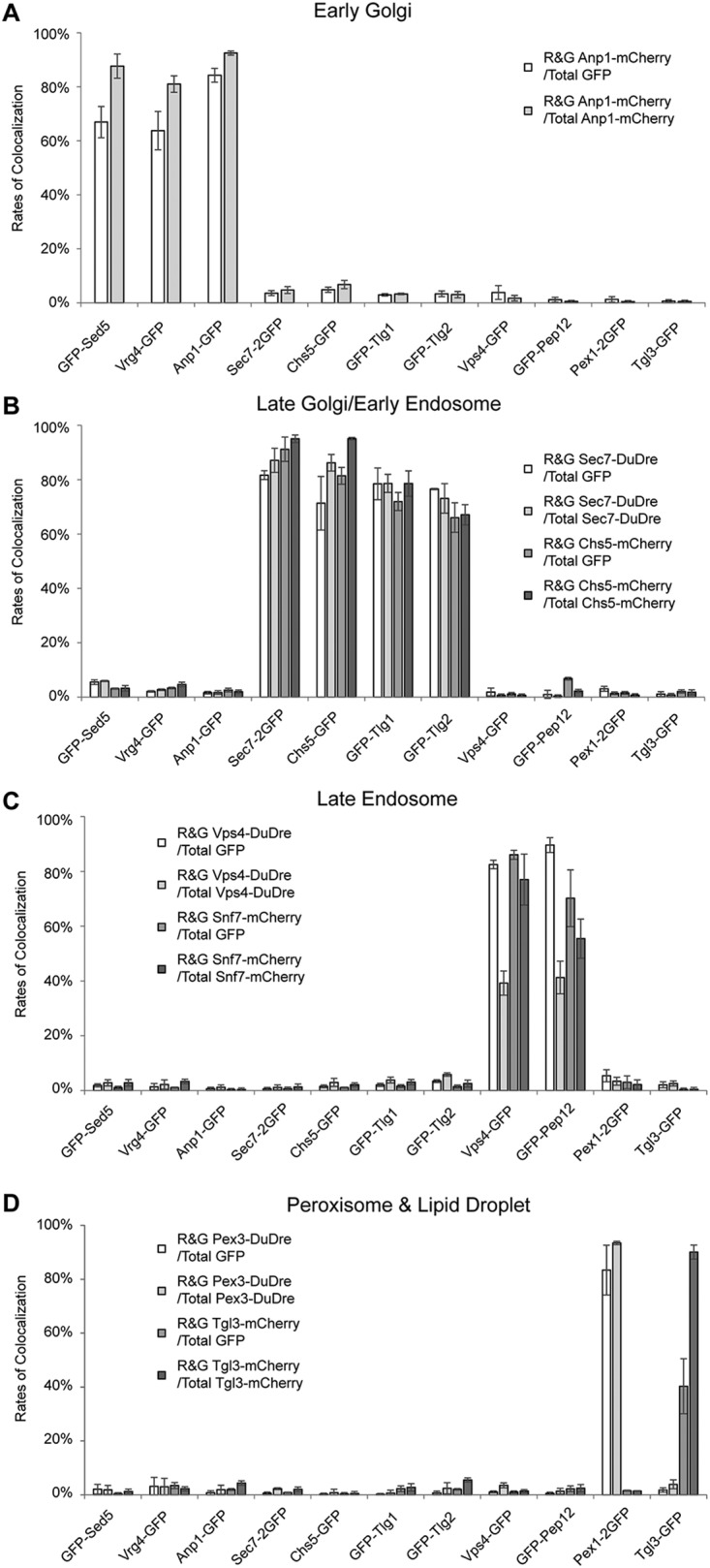
Quantification of colocalization among red and green punctate organelle markers. (A to D) Colocalization between red markers of early Golgi (A), late Golgi/early endosomes (B), late endosomes (C), and peroxisomes and lipid droplets (D) and green markers of these organelles were manually quantified. For each pair of red and green markers, two colocalization ratios were reported, one using the total number of green structures as the denominator and the other using red structures. Each experiment was repeated three times; each time, approximately 30 cells were analyzed. Error bar, standard deviation; *n* = 3.

Quantification of colocalization among markers of punctate signal patterns revealed that most markers of the same organelles have colocalization rates in the range of 76% ± 15% (mean ± standard deviation) (Fig. 8). In contrast, the rates among markers of different organelles are mostly in the range of 2% ± 2%. The two groups of numbers are in completely different leagues, confirming that the major organelles as we know are distinct entities (with the exception of late Golgi/early endosome). Another way to look at the numbers is that we can predict the tolerance intervals with 95% certainty that 95% of the ratios for markers of the same organelles will be above 44%, and those for different organelles will be below 5%. Any marker constructed in the future satisfying both parameters can be considered specific for a certain organelle.

Note that for each pair of markers, we reported two colocalization ratios, one with the number of the green dots as the denominator and the other with the red ones. The difference between the two results therefore reflects the difference in the two denominators, with the ones for Vps4-DuDre displaying the largest jump (Fig. 8C). In this case, the good signal of Vps4-DuDre allowed us to discern a lot more dots. Eighty-three percent of Vps4-GFP dots are Vps4-DuDre positive, whereas only 39% of Vps4-DuDre dots are Vps4-GFP positive. Despite the substantial increase in observable number, Vps4-DuDre dots did not colocalize with markers of other organelles (Fig. 4D and 8C). If we only use the ratio number with the dimmer/fewer one as the denominator, the colocalization rates for markers of the same organelles fall within 67% to 95%, with most in the 76% to 93% range.

### Functional analysis.

To assess the impact of marker expression on intracellular protein trafficking and organelle function, we employed the following assays: (i) a pulse-chase assay tracking the glycosylation status of a secretory cargo (Fig. S1), (ii) a protein endocytosis assay (Fig. S2), (iii) a growth assay to evaluate mitochondrial metabolic activity (Fig. S3A), (iv) a mitochondrial protein import assay (Fig. S3B), and (v) a growth assay to evaluate peroxisomal metabolic activity (Fig. S3C). The first two assays cover most organelles in the secretory pathway and endocytic pathway. The last three focus on mitochondria and peroxisomes, which are not directly positioned in these two pathways.

10.1128/mBio.01691-19.1 FIG S1 Functional analysis of organelle markers related to the secretory pathway. (A and B) The transit of Wsc1 in the secretory pathway was assessed by pulse-chase analysis in strains expressing the indicated chimeras either as the sole copy of the tagged protein (A) or as an extra to the endogenous copy (B). Markers of the ER, early Golgi, and late Golgi/early endosome were examined. Wsc1’s molecular weight increase after synthesis reflects the progression of glycosylation as the protein travels. The experiment was repeated three times. Representative images are shown. Download FIG S1, PDF file, 0.2 MB. Copyright © 2019 Zhu et al. 2019 Zhu et al. This content is distributed under the terms of the Creative Commons Attribution 4.0 International license.

10.1128/mBio.01691-19.2 FIG S2 Functional analysis of organelle markers related to endocytosis. (A and B) Lysine-induced endocytosis and turnover of Lyp1 were assessed by Western blotting in strains expressing the indicated chimeras either as the sole copy of the tagged protein (A) or as an extra to the endogenous copy (B). Markers of the late Golgi/early endosome, late endosome, and vacuole were examined. The experiment was repeated three times, with representative images shown. For ease of comparison, samples for each chimera-expressing strain were accompanied by a set of wild-type samples on the same membrane blot. Therefore, the images of some wild-type samples were reused. Download FIG S2, PDF file, 0.3 MB. Copyright © 2019 Zhu et al. 2019 Zhu et al. This content is distributed under the terms of the Creative Commons Attribution 4.0 International license.

10.1128/mBio.01691-19.3 FIG S3 Functional analysis of mitochondria and peroxisome markers. (A) The respiratory function of mitochondria was assessed by growth on no-fermentable carbon sources, ethanol and glycerol. Serial dilutions of cells were spotted on YPEG plates and incubated for 4 days. (B) Import of mitochondrial inner membrane protein, Atm1, was assessed by subcellular fractionation. All, total cell lysate; S, cytoplasmic supernatant fraction; P, pellet fraction containing mitochondria. (C) The metabolic function of peroxisomes was assessed by growth with oleic acid as the carbon source. Serial dilutions of cells were spotted on YPO plates and incubated for 4 days. In all experiments, representative images from 3 independent repeats are shown. FP, fluorescent protein. Download FIG S3, PDF file, 0.3 MB. Copyright © 2019 Zhu et al. 2019 Zhu et al. This content is distributed under the terms of the Creative Commons Attribution 4.0 International license.

Most of our marker constructs were designed to be integrated as an additional copy in the genome (Table 2). We therefore performed two sets of tests. One set utilized strains expressing the marker constructs as the sole copy (by either knock-in replacement or deleting the endogenous copy; see Materials and Methods for details). The other set utilized strains constructed as designed, carrying marker constructs as the second copy.

**TABLE 2:** Restriction sites or PCR primers used to generate linear fragments for yeast transformation

Plasmid (selection condition)	Restriction site(s) or PCR primer(s)^*a*^	Insert locus
Emc1-2GFP (Ura)	XhoI	*EMC1* ORF
pTPI1-GFP-HDEL (Ura)	PacI	*TPI1* promoter
Elo3-mCherry (Trp)	BstBI	*ELO3* promoter
pTPI1-mCherry-HDEL (Trp)	PacI	*TPI1* promoter
Sec63-2mTagBFP2 (Trp)	MfeI	*SEC63* ORF
Elo3-mTagBFP2 (Trp)	BstBI	*ELO3* promoter
Nab2-GFP (Ura)	KasI	*NAB2* ORF
Nab2-mCherry (Trp)	AvrII	*NAB2* promoter
Nab2-mTagBFP2 (Trp)	AgeI	*NAB2* ORF
GFP-Sed5 (Ura)	StuI and SnaBI	*ura3*
Vrg4-GFP (Ura)	BstBI	*VRG4* ORF
Anp1-GFP (Ura)	KpnI	*ANP1* ORF
Anp1-mCherry (Trp)	MfeI	*ANP1* ORF
Mnn9-mTagBFP2 (Trp)	EcoRI	*MNN9* ORF
Sec26-mTagBFP2 (Trp)	AfeI	*SEC26* ORF
Chs5-GFP (Ura)	AflII	*CHS5* promoter
Sec7-2GFP (Ura)	SnaBI and AflII^*b*^	*SEC7*
pCUP1-GFP-Tlg1 (Ura)	StuI	*TLG1* ORF
pCUP1-GFP-Tlg2 (Ura)	StuI	*TLG2* ORF
Sec7-DuDre (Trp)	KasI	*SEC7* promoter
Chs5-mCherry (Trp)	AflII	*CHS5* promoter
Sec7-mTagBFP2 (Trp)	NgoMIV	*SEC7* ORF
mTagBFP2-Tlg1 (Trp)	AflII	*TLG1* promoter
Vps4-GFP (Ura)	AfeI	*VPS4* ORF
GFP-Pep12 (Ura)	BS-UraF/BS-UraR	*ura3*
Vps4-DuDre (Trp)	MfeI	*VPS4* promoter
Snf7-mCherry (Trp)	MfeI	*SNF7* promoter
Vps4-mTagBFP2 (Trp)	AfeI	*VPS4* ORF
pCUP1-GFP-Pho8 (Ura)	StuI	*PHO8* ORF
Vph1-2GFP (Ura)	NheI and StuI^*b*^	*VPH1*
Vph1-mCherry (Trp)	PmlI	*VPH1* promoter
Vph1-mTagBFP2 (Trp)	EcoRI	*VPH1* ORF
Cox4-GFP (Ura)	BstBI	*COX4* promoter
Cox4-DuDre (Trp)	BsrGI	*COX4* promoter
Cox4-mTagBFP2 (Trp)	BsaBI	*COX4* promoter
Cox9-mTagBFP2 (Trp)	MfeI	*COX9* promoter
Pex1-2GFP (Ura)	AflII	*PEX1* ORF
Pex3-DuDre (Trp)	BsrGI	*PEX3* ORF
pTPI1-mTagBFP2-SKL (Trp)	PacI	*TPI1* promoter
Tgl3-GFP (Ura)	SnaBI	*TGL3* ORF
Tgl3-mCherry (Trp)	MfeI	*TGL3* promoter
Erg6-mCherry (Trp)	AgeI	*ERG6* ORF

a PCR primers are underlined.

b Linearizing these two plasmids as described here results in 3′ knock-in of the fluorescent protein and selection marker cassettes.

10.1128/mBio.01691-19.10 TABLE S2 Construction of plasmids and strains. (A) Brief descriptions of plasmid construction. (B) DNA sequences of PCR primers. Download Table S2, DOCX file, 0.1 MB. Copyright © 2019 Zhu et al. 2019 Zhu et al. This content is distributed under the terms of the Creative Commons Attribution 4.0 International license.

Wsc1 is a plasma membrane glycoprotein. Its transit through the secretory pathway is accompanied by changes in its glycosylation level (47). We examined the rates of its glycosylation in strains expressing markers of the ER, early Golgi, and late Golgi/early endosome (Fig. S1). By 20 min into the chase, most synthesized Wsc1 proteins were in the fully glycosylated form in all strains tested, indicating that these markers are functional.

Lyp1 is a lysine permease. In lysine-free medium, it is present on the plasma membrane. The addition of lysine triggers its endocytosis and eventual degradation in the vacuole (48). The rate of Lyp1 turnover was analyzed in strains expressing markers of late Golgi/early endosome, late endosome, and vacuole. The only strain in which we noticed a significant delay in Lyp1 turnover was the one expressing Snf7-mCherry as the sole copy (Fig. S2A). The turnover rate was normal when the endogenous copy of Snf7 was present (Fig. S2B). These results indicate that Snf7-mCherry is not fully functional by itself but does not impose any dominant negative effect. As mentioned previously, both Snf7-mCherry and Vps4-DuDre were more concentrated on the endosomes than were their GFP counterparts (Fig. 4A to C). Nevertheless, the function of Vps4-DuDre appeared to be normal in this particular assay.

To evaluate the metabolic functions of mitochondria and peroxisomes, we examined the growth of marker-expressing strains on agar plates containing ethanol and glycerol or oleic acid as the carbon source (49, 50). Strains carrying Cox4-GFP, Cox4-DuDre, Pex1-2GFP, and Pex3-DuDre all displayed growth rates similar to that of the wild-type strain (Fig. S3A and C). Furthermore, we examined mitochondrial protein import by evaluating Atm1 fractionation with mitochondria (51, 52) (Fig. S3B). All strains expressing Cox4-GFP and Cox4-DuDre displayed efficient import. These results demonstrate that our green and red markers for these two organelles are functional.

### The blues.

Compared with red fluorescent proteins, the current choices of blue fluorescent proteins are rather limited (53, 54). Only mTagBFP2 produced satisfactory signal intensity in our hands. When a red chimera mislocalized, we were sometimes able to solve the problem by switching to a different red fluorescent protein (Table S1). However, in the case of blue markers, we had to at times settle with less satisfactory performance. In particular, one issue we encountered with mTagBFP2 constructs was that a large number of them had a propensity to associate with the plasma membrane. Even more puzzling was the fact that this phenomenon was not stable. A construct might show plasma membrane signal in one experiment but behave perfectly normally in another experiment. So far, we were unable to pinpoint the underlying cause, except that it only occurred with mTagBFP2 constructs. As the plasma membrane is easily distinguishable from intracellular organelles, we consider the blue constructs to be somewhat usable and provide a brief description of them.

Similar to procedures we adopted for red markers, for large organelles with distinct morphological features, the fidelity of the blue constructs was verified by colocalization with green markers of the same intended organelles (or with organelle-specific dyes when available). For this group, we found the following markers to perform well (Fig. S4): (i) for the ER, Sec63-2magBFP2 and Elo3-mTagBFP2 (55); (ii) for the nucleus, Nab2-mTagBFP2; (iii) for vacuoles, Vph1-mTagBFP2; and (iv) for mitochondria, Cox4-mTagBFP2 and Cox9-mTagBFP2.

10.1128/mBio.01691-19.4 FIG S4 Blue markers of endoplasmic reticulum, nucleus, vacuoles, and mitochondria. Images were captured and presented as in Fig. 1, except that blue fluorescent protein fusions were pseudocolored red in colocalization images. (A) Blue markers of ER. (B) Colocalization between green and blue ER markers. (C) A blue marker of the nucleus. (D) Colocalization between green and blue nucleus markers. (E) A blue marker of vacuoles. (F) Colocalization between green and blue vacuole markers. (G) Blue markers of mitochondria. (H) Colocalization between green and blue mitochondria markers. Download FIG S4, PDF file, 1.1 MB. Copyright © 2019 Zhu et al. 2019 Zhu et al. This content is distributed under the terms of the Creative Commons Attribution 4.0 International license.

For organelles with scattered punctate distribution, additional cross-comparisons with other organelles were carried out. The current list of usable markers includes (i) for early Golgi, Sec26-mTagBFP2 and Mnn9-mTagBFP2 (Fig. S5) (24, 56); (ii) for late Golgi/early endosomes, Sec7-mTagBFP2 and mTagBFP2–Tlg1 (Fig. S6); (iii) for late endosomes, Vps4-mTagBFP2 (Fig. S7A to C); and (iv) for peroxisomes, mTagBFP2-SKL (Fig. S7D and E). As the signal of Mnn9-mTagBFP2 was weaker than the others, a large difference between the pairs of colocalization ratios was observed (Fig. S8). Similar to the Vps4-DuDre case discussed above, no substantial colocalization between Mnn9-mTagBFP2 and markers of other organelles was observed. Notably, Vps4-mTagBFP2 displayed partial colocalization with green markers of late Golgi/early endosomes (Fig. S7C and S8C). We tested eight other candidate late endosomal proteins, and none performed better (Table S1). In the case of lipid droplets, although Erg6-mTagBFP2 displayed good signal, it appeared to aggregate and substantially reduce the number of lipid droplets (Table S1).

10.1128/mBio.01691-19.5 FIG S5 Blue markers of early Golgi. Images were captured and presented as in Fig. S4. (A) Blue markers of early Golgi. (B) Colocalization between green and blue early Golgi markers. (C) Lack of colocalization between green markers of other organelles and blue markers of early Golgi. Download FIG S5, PDF file, 2.6 MB. Copyright © 2019 Zhu et al. 2019 Zhu et al. This content is distributed under the terms of the Creative Commons Attribution 4.0 International license.

10.1128/mBio.01691-19.6 FIG S6 Blue markers of late Golgi/early endosomes. Images were captured and presented as in Fig. S4. (A) Blue markers of late Golgi/early endosomes. (B) Colocalization between green and blue late Golgi/early endosome markers. (C) Lack of colocalization between green markers of other organelles and blue markers of late Golgi/early endosomes. Download FIG S6, PDF file, 2.6 MB. Copyright © 2019 Zhu et al. 2019 Zhu et al. This content is distributed under the terms of the Creative Commons Attribution 4.0 International license.

10.1128/mBio.01691-19.7 FIG S7 Blue markers of late endosomes and peroxisomes. Images were captured and presented as in Fig. S4. (A) A blue marker of late endosomes. (B) Colocalization between green and blue late endosome markers. (C) Cross-comparison between green markers of other organelles and the blue marker of late endosomes. Some Vps4-mTagBFP2 puncta colocalized with late Golgi/early endosome green markers. (D) A blue marker of peroxisomes. (E) Colocalization between green and blue peroxisome markers. (F) Lack of colocalization between green markers of other organelles and the blue marker of peroxisomes. Download FIG S7, PDF file, 2.3 MB. Copyright © 2019 Zhu et al. 2019 Zhu et al. This content is distributed under the terms of the Creative Commons Attribution 4.0 International license.

10.1128/mBio.01691-19.8 FIG S8 Quantification of colocalization among blue and green punctate organelle markers. (A to D) Colocalization between blue markers of early Golgi (A), late Golgi/early endosomes (B), late endosomes (C), and peroxisomes (D) and green markers of these organelles were manually quantified and presented as in Fig. 8. Download FIG S8, PDF file, 0.1 MB. Copyright © 2019 Zhu et al. 2019 Zhu et al. This content is distributed under the terms of the Creative Commons Attribution 4.0 International license.

Wsc1 pulse-chase and Lyp1 endocytosis assays demonstrated that all blue markers of the ER, early Golgi, late Golgi/early endosome, and vacuole are functional (Fig. S1 and S2). Mitochondrial protein import and peroxisomal metabolic activity were also normal in strains expressing the blue markers (Fig. S3). However, when Cox9-mTagBFP was expressed as the sole copy, growth on glycerol plates was retarded, indicating that Cox9-mTagBFP is only partially functional in respiration (Fig. S3). Growth was normal when endogenous Cox9 was present.

## DISCUSSION

In this study, we constructed fluorescent protein chimeras of candidate proteins and systematically evaluated their performance as organelle markers in live yeast cells. Our markers include three colors and cover the ER, nucleus, Golgi apparatus, endosomes, vacuoles, mitochondria, peroxisomes, and lipid droplets. As noted in Table S1, some of the candidate proteins considered for use as organelle markers in the existing literature localize to more than one organelle or even completely mislocalize when fused with certain fluorescent proteins. The availability of our marker set can potentially help clarify inappropriate interpretations of data arising from the use of leaky or faulty markers. An important message we would like to emphasize, however, is that the data reported here are applicable to and only to the exact constructs. The purpose of this work is to obtain a set of markers that are “good enough.” We therefore did not make extensive attempts to optimize individual designs or to create exact duplicates of previously reported constructs. Subtle differences in linker sequences and other design choices certainly can change the localization of otherwise similar chimeras, which might explain the variation in the final results.

Historically, most organelles as we know them today were discovered and defined using data from chemical staining, electron microscopy, subcellular fractionation, and biochemical characterizations. In the age of digital live-cell imaging, fluorescent-protein-based labeling is often the first tool of choice for cell biology investigators. It is therefore critical to have fact-based criteria to define organelle identities and protein localization from the fluorescent protein imaging perspective. Our quantification revealed that most chimera markers for the same organelles have a colocalization rate above 61%, with the average being around 76% (Fig. 8). Perhaps more important is that the colocalization rates among markers of distinct organelles are all below 7%, with the average being around 2% (Fig. 8). These numbers provides reference points for the objective definition of “full/complete” or “no” colocalization, which are otherwise only of descriptive value.

In addition, one incidental outcome of our study was the realization that late Golgi and early endosomes, two terms with seemly distinct identities, denote the same compartment at the resolution of light microscopy. The confusion has lingered around for some time, and both early and current publications have expressed the difficulty in separating the two using fluorescent protein markers (57–60). A recent in-depth study by Day et al. delivered a compelling case supporting their consolidation in yeast (7). The same compartment has also been designated “chitosomes” in some literature (57). In our case, Sec7-2GFP, Chs5-GFP, GFP-Tlg1, and GFP-Tlg2 all displayed high ratios of colocalization with Sec7-DuDre and Chs5-mCherry in the 66 to 96% range, in contrast to the sub-7% ratios with all other compartments (Fig. 8). Their colocalization ratios with FM4-64 are also comparable, ranging from 58% to 86%. The numbers from Sec7-mTagBFP2 and mTagBFP2-Tlg1 painted a similar picture (Fig. S8). For any open-minded observer, these numbers speak volumes.

Retrospectively, our marker validation process constituted an ideal opportunity to assess the current organelle nomenclature. An imminent question is whether any additional case of nomenclature consolidation awaits our contemplation. In this regard, we report with confidence and delight that that no other major organelle is missing in yeast, and therefore, general insights gained from yeast intracellular trafficking studies remain highly relevant for other eukaryotes.

Last, it should be noted that intracellular trafficking is a highly dynamic process subject to regulation by environmental factors (61, 62). In some cases, it can result in drastic changes in the subcellular localization of a protein. For example, the presence of methionine not only inactivates the expression of methionine transporter Mup1 but also triggers its translocation from the plasma membrane to the vacuolar lumen (63). The Lyp1 reporter we utilized is another example of this type. Our microscopy observations were performed with mid-log-phase yeast cells incubated in liquid culture, which represents a healthy low-stress state. It is conceivable that under certain experimental conditions, some of the marker proteins may relocate somewhere else instead of sites confirmed in the present study, and one may need to search for a replacement in its place. This is another reason why we included a description of the fluorescent protein chimeras that did not work well for us (Table S1). These constructs might suit the needs of colleagues looking to investigate other questions.

## MATERIALS AND METHODS

### Plasmids and strains.

Plasmid construction was performed using common procedures involving insert fragment amplification by PCR, restriction digestion, and ligation. The restriction sites and PCR primers utilized are listed in Table S2A. The DNA sequences of the primers are listed in Table S2B.

For most single-/dual-color single-time-point imaging experiments, plasmids containing marker proteins were transformed into TN124 (*MAT***a** *leu2 ura3 trp1 pho8*Δ*60 pho13*Δ::*LEU2*) (64). For tricolor time-lapse imaging, plasmids containing marker proteins were transformed into DJ03 (BY4741 *trp1*Δ::*MET15*) (65). Functional analysis was performed in YZJ033 (BY4741 *trp1*Δ::*nat*)-derived strains.

Prior to transformation, linear fragments were obtained either through restriction digestion or PCR amplification. Among the plasmids constructed, most are designed to be integrated as an additional copy. Only Sec7-2GFP and Vph1-2GFP are designed as 3′-knock-in constructs. The restriction sites and primers utilized to generate linear fragments as designed are listed in Table 2.

To evaluate protein function with the chimeras as the sole copy of the target gene, we also constructed a second set of strains, mostly using PCR to generate linear knock-in fragments. The sequences of these primers are listed in Table S2B, with “recombination” in their names. Three exceptions are GFP-Sed5, GFP-Pep12, and mTagBFP2-Tlg1. For these strains, the chimeras were integrated as an extra copy, with the endogenous open reading frames (ORFs) knocked out in a separate step.

### Yeast media.

The yeast media used were SMD (2% glucose, 0.67% yeast nitrogen base without amino acids, 30 mg/liter adenine, 30 mg/liter lysine, 30 mg/liter methionine, 20 mg/liter histidine, 20 mg/liter uracil, 50 mg/liter tryptophan, 50 mg/liter leucine), SMD + CA (SMD with the addition of 0.5% Casamino Acids), SD + Trp (2% glucose, 0.67% yeast nitrogen base without amino acids, 50 mg/liter tryptophan), YPR (1% yeast extract, 2% peptone, 2% raffinose), YPD (1% yeast extract, 2% peptone, 2% glucose), YPEG (1% yeast extract, 2% peptone, 3% ethanol, and 3% glycerol), and YPO (1% yeast extract, 2% peptone, 0.2% oleic acid, and 0.02% Tween 80).

### Fluorescence microscopy.

Unless otherwise noted, yeast cells were cultured in SMD medium at 30°C. Colonies were first inoculated into liquid culture. On the morning of the second day, the optical density at 600 nm (OD_600_) of the culture was adjusted to approximately 0.2. Upon reaching an OD_600_ of approximately 0.8 to 1.0, 200 μl of liquid culture per sample was collected and allowed to precipitate on concanavalin A-coated cover glass for 5 min. Image stacks (15 slices, 0.5-μm step size) were collected on an Olympus IX83 inverted fluorescence microscope equipped with a Hamamatsu Orca Flash4.0 LT camera and a Lumencor Spectra X six-channel light source. For all single-time-point image stack captures, the intensity of the excitation was set to 100%, and the exposure time for each frame was 100 ms (for GFP) or 200 ms (for red fluorescent protein [RFP] and blue fluorescent protein [BFP]).

For time-lapse imaging, cells were immobilized on concanavalin A-coated glass-bottom dishes and incubated in SMD + CA medium at 30°C. Image stacks were collected every 10 min. A median filter (ImageJ – process – noise – despeckle) was applied to reduce noise (66, 67).

For FM4-64 staining of endocytic compartments, 2 ml of liquid culture with an OD_600_ of approximately 0.8 to 1.0 was collected and resuspended in ice-cold SMD medium containing 16 μM FM4-64. After incubation on ice for 15 min, cells were washed with ice-cold SMD medium 3 times. Cells were then resuspended in 2 ml SMD medium and incubated at 15°C for 10 min. At this moment, image stacks were collected as described above.

For DAPI (nucleus) and BODIPY (lipid droplet) staining, 2 μl dye (5 mg/ml DAPI or 0.01 mg/ml BODIPY) was added to 100 μl of yeast liquid culture, and the mixture was left to sediment on concanavalin A-coated cover glass for 5 min. After washing with fresh medium, adhered yeast cells were observed under microscope. For MitoTracker red (mitochondria) staining, 0.1 μl dye (1 mM) was added to 2 ml yeast culture to stain for 5 min. After washing with fresh medium, yeast cells were attached to concanavalin A-coated cover glass for observation.

### Quantification of colocalization ratios.

Each time, a region of approximately 700 by 700 pixels was chosen, which contained around 30 cells. The total number of dots in each color channel was counted first, generating the denominators. For dots suspected to colocalize, each case was further examined to determine (i) if the dots are in the same z-section, and (ii) if the areas covered by the two dots overlap by more than 50%. Cases meeting both criteria were categorized as colocalizing, generating the nominator.

### Wsc1 pulse-chase analysis.

Strains to be tested were transformed with two additional plasmids, pEcOmeTyr/ectRNA_CUA_ (MET) and Yeplac181-GAL1-10-tc-apta-amber*-WSC1-FPA (LEU) (47).

Yeast cells were initially inoculated into SD-Met-Leu medium to grow overnight. On the second day, yeast cells were transferred to YPR medium, with a starting OD_600_ of 0.2. Upon reaching an OD_600_ of 0.5, 2% galactose was added to induce mRNA transcription for 20 min. This was followed by the addition of 1 mM Ome-Tyr to allow for translation of the full-length protein for 5 min. Yeast cells were then transferred to YPD medium containing 350 μg/ml tetracycline to initiate the chase stage. Time 0 samples were collected right before the chase medium transfer. Time 6 and 20 samples were collected 6 and 20 min after the transfer, respectively. Samples were analyzed by Western blotting using common procedures.

### Lyp1 endocytosis assay.

In strains to be tested, Lyp1 was further tagged at the C terminus with 9MYC. The strains were then transformed with the pHULM plasmid to become prototrophic (68).

Yeast cells were initially inoculated into SD + Trp medium to grow overnight. On the second day, yeast cells first diluted to an OD_600_ of 0.2. Upon reaching an OD_600_ of 0.8, 230 μg/ml lysine was added to the medium. Samples were collected at 0, 1, and 3 h after the addition of lysine and analyzed by Western blotting.

### YPEG and YPO growth assays.

1 OD_600_ of log phase yeast cells were suspended in 1 ml water, from which 10× serial dilutions were prepared. Four microliters of suspension was spotted onto YPEG or YPO agar plates and incubated at 30°C for 4 days.

### Mitochondrial protein import assay.

In strains to be tested, the mitochondrial inner membrane protein Atm1 was further tagged at the C terminus with 3-hemagglutinin (3HA). Lyticase was prepared from Escherichia coli strain RSB805 (69).

Yeast cells were grown to mid-log phase. 15 OD_600_ of cells were collected, washed in 0.1 M Tris-HCl (pH 9.4), suspended in 15 mM dithiothreitol (DTT) and 0.1 M Tris-HCl (pH 9.4), and incubated at 30°C for 15 min. Afterwards, cells were transferred to spheroplast buffer [20 mM 20 mM piperazine-*N*,*N*′-bis(2-ethanesulfonic acid) PIPES (pH 6.8), 1 M sorbitol, 1/5 lyticase prep] and incubated at 30°C for 30 min. Spheroplasts were collected by centrifugation at 1,000 × *g* and 4°C for 5 min, resuspended in 1 ml ice-cold lysis buffer (PIPES [pH 6.8], 50 mM KOAc, 150 mM NaCl, 600 mM sorbitol, 1 mM EDTA, 1 mM phenylmethylsulfonyl fluoride [PMSF]), and lysed by 15 strokes in a glass homogenizer placed on ice. Cell debris was removed by centrifugation at 1,700 × *g* and 4°C for 5 min. The lysate (S1700 supernatant) was further centrifuged at 17,000 × *g* and 4°C for 12 min to obtain the cytoplasmic fraction (S17000 supernatant). The pellet was washed in lysis buffer and centrifuged again to obtain the mitochondrial fraction (P17000). The distribution of Atm1 between the cytoplasmic fraction and the mitochondrial fraction was analyzed by Western blotting.

## References

[1] Elias M 2010 Patterns and processes in the evolution of the eukaryotic endomembrane system. Mol Membr Biol 27:469–489. doi:10.3109/09687688.2010.521201. 21067450

[2] Schlacht A, Herman EK, Klute MJ, Field MC, Dacks JB 2014 Missing pieces of an ancient puzzle: evolution of the eukaryotic membrane-trafficking system. Cold Spring Harb Perspect Biol 6:a016048. doi:10.1101/cshperspect.a016048. 25274701 PMC4176009

[3] Levine B, Klionsky DJ 2017 Autophagy wins the 2016 Nobel Prize in physiology or medicine: breakthroughs in baker’s yeast fuel advances in biomedical research. Proc Natl Acad Sci U S A 114:201–205. doi:10.1073/pnas.1619876114. 28039434 PMC5240711

[4] Yuan W, Veenhuis M, van der Klei IJ 2016 The birth of yeast peroxisomes. Biochim Biophys Acta 1863:902–910. doi:10.1016/j.bbamcr.2015.09.008. 26367802

[5] Schekman R 2010 Charting the secretory pathway in a simple eukaryote. Mol Biol Cell 21:3781–3784. doi:10.1091/mbc.E10-05-0416. 21079008 PMC2982102

[6] Dimitrov L, Lam SK, Schekman R 2013 The role of the endoplasmic reticulum in peroxisome biogenesis. Cold Spring Harb Perspect Biol 5:a013243. doi:10.1101/cshperspect.a013243. 23637287 PMC3632059

[7] Day KJ, Casler JC, Glick BS 2018 Budding yeast has a minimal endomembrane system. Dev Cell 44:56–72.e54. doi:10.1016/j.devcel.2017.12.014. 29316441 PMC5765772

[8] Day RN, Davidson MW 2009 The fluorescent protein palette: tools for cellular imaging. Chem Soc Rev 38:2887–2921. doi:10.1039/b901966a. 19771335 PMC2910338

[9] Lee S, Lim WA, Thorn KS 2013 Improved blue, green, and red fluorescent protein tagging vectors for S. cerevisiae. PLoS One 8:e67902. doi:10.1371/journal.pone.0067902. 23844123 PMC3699464

[10] Moore I, Murphy A 2009 Validating the location of fluorescent protein fusions in the endomembrane system. Plant Cell 21:1632–1636. doi:10.1105/tpc.109.068668. 19561167 PMC2714940

[11] Stadler C, Rexhepaj E, Singan VR, Murphy RF, Pepperkok R, Uhlen M, Simpson JC, Lundberg E 2013 Immunofluorescence and fluorescent-protein tagging show high correlation for protein localization in mammalian cells. Nat Methods 10:315–323. doi:10.1038/nmeth.2377. 23435261

[12] West M, Zurek N, Hoenger A, Voeltz GK 2011 A 3D analysis of yeast ER structure reveals how ER domains are organized by membrane curvature. J Cell Biol 193:333–346. doi:10.1083/jcb.201011039. 21502358 PMC3080256

[13] Lahiri S, Chao JT, Tavassoli S, Wong AK, Choudhary V, Young BP, Loewen CJ, Prinz WA 2014 A conserved endoplasmic reticulum membrane protein complex (EMC) facilitates phospholipid transfer from the ER to mitochondria. PLoS Biol 12:e1001969. doi:10.1371/journal.pbio.1001969. 25313861 PMC4196738

[14] Toke DA, Martin CE 1996 Isolation and characterization of a gene affecting fatty acid elongation in Saccharomyces cerevisiae. J Biol Chem 271:18413–18422. doi:10.1074/jbc.271.31.18413. 8702485

[15] Merzlyak EM, Goedhart J, Shcherbo D, Bulina ME, Shcheglov AS, Fradkov AF, Gaintzeva A, Lukyanov KA, Lukyanov S, Gadella TW, Chudakov DM 2007 Bright monomeric red fluorescent protein with an extended fluorescence lifetime. Nat Methods 4:555–557. doi:10.1038/nmeth1062. 17572680

[16] Semenza JC, Hardwick KG, Dean N, Pelham HR 1990 ERD2, a yeast gene required for the receptor-mediated retrieval of luminal ER proteins from the secretory pathway. Cell 61:1349–1357. doi:10.1016/0092-8674(90)90698-e. 2194670

[17] Anderson JT, Wilson SM, Datar KV, Swanson MS 1993 NAB2: a yeast nuclear polyadenylated RNA-binding protein essential for cell viability. Mol Cell Biol 13:2730–2741. doi:10.1128/mcb.13.5.2730. 8474438 PMC359649

[18] Losev E, Reinke CA, Jellen J, Strongin DE, Bevis BJ, Glick BS 2006 Golgi maturation visualized in living yeast. Nature 441:1002–1006. doi:10.1038/nature04717. 16699524

[19] Matsuura-Tokita K, Takeuchi M, Ichihara A, Mikuriya K, Nakano A 2006 Live imaging of yeast Golgi cisternal maturation. Nature 441:1007–1010. doi:10.1038/nature04737. 16699523

[20] Burri L, Lithgow T 2004 A complete set of SNAREs in yeast. Traffic 5:45–52. doi:10.1046/j.1600-0854.2003.00151.x. 14675424

[21] Søgaard M, Tani K, Ye RR, Geromanos S, Tempst P, Kirchhausen T, Rothman JE, Sollner T 1994 A rab protein is required for the assembly of SNARE complexes in the docking of transport vesicles. Cell 78:937–948. doi:10.1016/0092-8674(94)90270-4. 7923363

[22] Sacher M, Stone S, Ferro-Novick S 1997 The synaptobrevin-related domains of Bos1p and Sec22p bind to the syntaxin-like region of Sed5p. J Biol Chem 272:17134–17138. doi:10.1074/jbc.272.27.17134. 9202032

[23] Dean N, Zhang YB, Poster JB 1997 The VRG4 gene is required for GDP-mannose transport into the lumen of the Golgi in the yeast, Saccharomyces cerevisiae. J Biol Chem 272:31908–31914. doi:10.1074/jbc.272.50.31908. 9395539

[24] Jungmann J, Munro S 1998 Multi-protein complexes in the cis Golgi of Saccharomyces cerevisiae with alpha-1,6-mannosyltransferase activity. EMBO J 17:423–434. doi:10.1093/emboj/17.2.423. 9430634 PMC1170393

[25] Li D, Song JZ, Shan MH, Li SP, Liu W, Li H, Zhu J, Wang Y, Lin J, Xie Z 2015 A fluorescent tool set for yeast Atg proteins. Autophagy 11:954–960. doi:10.1080/15548627.2015.1040971. 25998947 PMC4502664

[26] Strack RL, Strongin DE, Bhattacharyya D, Tao W, Berman A, Broxmeyer HE, Keenan RJ, Glick BS 2008 A noncytotoxic DsRed variant for whole-cell labeling. Nat Methods 5:955–957. doi:10.1038/nmeth.1264. 18953349 PMC4107390

[27] Sata M, Donaldson JG, Moss J, Vaughan M 1998 Brefeldin A-inhibited guanine nucleotide-exchange activity of Sec7 domain from yeast Sec7 with yeast and mammalian ADP ribosylation factors. Proc Natl Acad Sci U S A 95:4204–4208. doi:10.1073/pnas.95.8.4204. 9539714 PMC22466

[28] Wang CW, Hamamoto S, Orci L, Schekman R 2006 Exomer: a coat complex for transport of select membrane proteins from the trans-Golgi network to the plasma membrane in yeast. J Cell Biol 174:973–983. doi:10.1083/jcb.200605106. 17000877 PMC2064389

[29] Trautwein M, Schindler C, Gauss R, Dengjel J, Hartmann E, Spang A 2006 Arf1p, Chs5p and the ChAPs are required for export of specialized cargo from the Golgi. EMBO J 25:943–954. doi:10.1038/sj.emboj.7601007. 16498409 PMC1409733

[30] Abeliovich H, Grote E, Novick P, Ferro-Novick S 1998 Tlg2p, a yeast syntaxin homolog that resides on the Golgi and endocytic structures. J Biol Chem 273:11719–11727. doi:10.1074/jbc.273.19.11719. 9565594

[31] Holthuis JC, Nichols BJ, Dhruvakumar S, Pelham HR 1998 Two syntaxin homologues in the TGN/endosomal system of yeast. EMBO J 17:113–126. doi:10.1093/emboj/17.1.113. 9427746 PMC1170363

[32] Wollert T, Yang D, Ren X, Lee HH, Im YJ, Hurley JH 2009 The ESCRT machinery at a glance. J Cell Sci 122:2163–2166. doi:10.1242/jcs.029884. 19535731 PMC2723142

[33] Burd C, Cullen PJ 2014 Retromer: a master conductor of endosome sorting. Cold Spring Harb Perspect Biol 6:a016774. doi:10.1101/cshperspect.a016774. 24492709 PMC3941235

[34] Babst M, Wendland B, Estepa EJ, Emr SD 1998 The Vps4p AAA ATPase regulates membrane association of a Vps protein complex required for normal endosome function. EMBO J 17:2982–2993. doi:10.1093/emboj/17.11.2982. 9606181 PMC1170638

[35] Becherer KA, Rieder SE, Emr SD, Jones EW 1996 Novel syntaxin homologue, Pep12p, required for the sorting of lumenal hydrolases to the lysosome-like vacuole in yeast. Mol Biol Cell 7:579–594. doi:10.1091/mbc.7.4.579. 8730101 PMC275911

[36] Gerrard SR, Levi BP, Stevens TH 2000 Pep12p is a multifunctional yeast syntaxin that controls entry of biosynthetic, endocytic and retrograde traffic into the prevacuolar compartment. Traffic 1:259–269. doi:10.1034/j.1600-0854.2000.010308.x. 11208109

[37] Babst M, Katzmann DJ, Estepa-Sabal EJ, Meerloo T, Emr SD 2002 Escrt-III: an endosome-associated heterooligomeric protein complex required for mvb sorting. Dev Cell 3:271–282. doi:10.1016/S1534-5807(02)00220-4. 12194857

[38] Bowers K, Stevens TH 2005 Protein transport from the late Golgi to the vacuole in the yeast Saccharomyces cerevisiae. Biochim Biophys Acta 1744:438–454. doi:10.1016/j.bbamcr.2005.04.004. 15913810

[39] Klionsky DJ, Emr SD 1989 Membrane protein sorting: biosynthesis, transport and processing of yeast vacuolar alkaline phosphatase. EMBO J 8:2241–2250. doi:10.1002/j.1460-2075.1989.tb08348.x. 2676517 PMC401154

[40] Toshima JY, Nishinoaki S, Sato Y, Yamamoto W, Furukawa D, Siekhaus DE, Sawaguchi A, Toshima J 2014 Bifurcation of the endocytic pathway into Rab5-dependent and -independent transport to the vacuole. Nat Commun 5:3498. doi:10.1038/ncomms4498. 24667230

[41] Manolson MF, Proteau D, Preston RA, Stenbit A, Roberts BT, Hoyt MA, Preuss D, Mulholland J, Botstein D, Jones EW 1992 The VPH1 gene encodes a 95-kDa integral membrane polypeptide required for in vivo assembly and activity of the yeast vacuolar H^+^-ATPase. J Biol Chem 267:14294–14303. 1385813

[42] Kaneko Y, Toh-e A, Oshima Y 1982 Identification of the genetic locus for the structural gene and a new regulatory gene for the synthesis of repressible alkaline phosphatase in Saccharomyces cerevisiae. Mol Cell Biol 2:127–137. doi:10.1128/mcb.2.2.127. 7050668 PMC369765

[43] Maréchal A, Meunier B, Lee D, Orengo C, Rich PR 2012 Yeast cytochrome c oxidase: a model system to study mitochondrial forms of the haem-copper oxidase superfamily. Biochim Biophys Acta 1817:620–628. doi:10.1016/j.bbabio.2011.08.011. 21925484 PMC4671319

[44] Gao Q, Goodman JM 2015 The lipid droplet–a well-connected organelle. Front Cell Dev Biol 3:49. doi:10.3389/fcell.2015.00049. 26322308 PMC4533013

[45] Ma C, Agrawal G, Subramani S 2011 Peroxisome assembly: matrix and membrane protein biogenesis. J Cell Biol 193:7–16. doi:10.1083/jcb.201010022. 21464226 PMC3082194

[46] Klug L, Daum G 2014 Yeast lipid metabolism at a glance. FEMS Yeast Res 14:369–388. doi:10.1111/1567-1364.12141. 24520995

[47] Stelter P, Kunze R, Radwan M, Thomson E, Thierbach K, Thoms M, Hurt E 2012 Monitoring spatiotemporal biogenesis of macromolecular assemblies by pulse-chase epitope labeling. Mol Cell 47:788–796. doi:10.1016/j.molcel.2012.06.015. 22819325

[48] Lin CH, MacGurn JA, Chu T, Stefan CJ, Emr SD 2008 Arrestin-related ubiquitin-ligase adaptors regulate endocytosis and protein turnover at the cell surface. Cell 135:714–725. doi:10.1016/j.cell.2008.09.025. 18976803

[49] Campbell CL, Thorsness PE 1998 Escape of mitochondrial DNA to the nucleus in yme1 yeast is mediated by vacuolar-dependent turnover of abnormal mitochondrial compartments. J Cell Sci 111:2455–2464. 9683639 10.1242/jcs.111.16.2455

[50] Thakur JK, Arthanari H, Yang F, Chau KH, Wagner G, Naar AM 2009 Mediator subunit Gal11p/MED15 is required for fatty acid-dependent gene activation by yeast transcription factor Oaf1p. J Biol Chem 284:4422–4428. doi:10.1074/jbc.M808263200. 19056732 PMC3837390

[51] Corral-Debrinski M, Belgareh N, Blugeon C, Claros MG, Doye V, Jacq C 1999 Overexpression of yeast karyopherin Pse1p/Kap121p stimulates the mitochondrial import of hydrophobic proteins in vivo. Mol Microbiol 31:1499–1511. doi:10.1046/j.1365-2958.1999.01295.x. 10200968

[52] Zischka H, Kinkl N, Braun RJ, Ueffing M 2008 Purification of Saccharomyces cerevisiae mitochondria by zone electrophoresis in a free flow device. Methods Mol Biol 432:51–64. doi:10.1007/978-1-59745-028-7_4. 18370010

[53] Subach OM, Cranfill PJ, Davidson MW, Verkhusha VV 2011 An enhanced monomeric blue fluorescent protein with the high chemical stability of the chromophore. PLoS One 6:e28674. doi:10.1371/journal.pone.0028674. 22174863 PMC3234270

[54] Ai HW, Shaner NC, Cheng Z, Tsien RY, Campbell RE 2007 Exploration of new chromophore structures leads to the identification of improved blue fluorescent proteins. Biochemistry 46:5904–5910. doi:10.1021/bi700199g. 17444659

[55] Mandon EC, Trueman SF, Gilmore R 2013 Protein translocation across the rough endoplasmic reticulum. Cold Spring Harb Perspect Biol 5:a013342. doi:10.1101/cshperspect.a013342. 23251026 PMC3552503

[56] Duden R, Hosobuchi M, Hamamoto S, Winey M, Byers B, Schekman R 1994 Yeast beta- and beta′-coat proteins (COP). Two coatomer subunits essential for endoplasmic reticulum-to-Golgi protein traffic. J Biol Chem 269:24486–24495. 7929113

[57] Valdivia RH, Baggott D, Chuang JS, Schekman RW 2002 The yeast clathrin adaptor protein complex 1 is required for the efficient retention of a subset of late Golgi membrane proteins. Dev Cell 2:283–294. doi:10.1016/S1534-5807(02)00127-2. 11879634

[58] Lewis MJ, Nichols BJ, Prescianotto-Baschong C, Riezman H, Pelham HR 2000 Specific retrieval of the exocytic SNARE Snc1p from early yeast endosomes. Mol Biol Cell 11:23–38. doi:10.1091/mbc.11.1.23. 10637288 PMC14754

[59] Robinson M, Poon PP, Schindler C, Murray LE, Kama R, Gabriely G, Singer RA, Spang A, Johnston GC, Gerst JE 2006 The Gcs1 Arf-GAP mediates Snc1,2 v-SNARE retrieval to the Golgi in yeast. Mol Biol Cell 17:1845–1858. doi:10.1091/mbc.e05-09-0832. 16452633 PMC1415299

[60] Weill U, Arakel EC, Goldmann O, Golan M, Chuartzman S, Munro S, Schwappach B, Schuldiner M 2018 Toolbox: creating a systematic database of secretory pathway proteins uncovers new cargo for COPI. Traffic 19:370–379. doi:10.1111/tra.12560. 29527758 PMC5947560

[61] van Leeuwen W, van der Krift F, Rabouille C 2018 Modulation of the secretory pathway by amino-acid starvation. J Cell Biol 217:2261–271. doi:10.1083/jcb.201802003. 29669743 PMC6028531

[62] Nardozzi JD, Lott K, Cingolani G 2010 Phosphorylation meets nuclear import: a review. Cell Commun Signal 8:32. doi:10.1186/1478-811X-8-32. 21182795 PMC3022542

[63] Menant A, Barbey R, Thomas D 2006 Substrate-mediated remodeling of methionine transport by multiple ubiquitin-dependent mechanisms in yeast cells. EMBO J 25:4436–4447. doi:10.1038/sj.emboj.7601330. 16977312 PMC1589980

[64] Noda T, Matsuura A, Wada Y, Ohsumi Y 1995 Novel system for monitoring autophagy in the yeast Saccharomyces cerevisiae. Biochem Biophys Res Commun 210:126–132. doi:10.1006/bbrc.1995.1636. 7741731

[65] Brachmann CB, Davies A, Cost GJ, Caputo E, Li J, Hieter P, Boeke JD 1998 Designer deletion strains derived from Saccharomyces cerevisiae S288C: a useful set of strains and plasmids for PCR-mediated gene disruption and other applications. Yeast 14:115–132. doi:10.1002/(SICI)1097-0061(19980130)14:2<115::AID-YEA204>3.0.CO;2-2. 9483801

[66] Schindelin J, Arganda-Carreras I, Frise E, Kaynig V, Longair M, Pietzsch T, Preibisch S, Rueden C, Saalfeld S, Schmid B, Tinevez JY, White DJ, Hartenstein V, Eliceiri K, Tomancak P, Cardona A 2012 Fiji: an open-source platform for biological-image analysis. Nat Methods 9:676–682. doi:10.1038/nmeth.2019. 22743772 PMC3855844

[67] Rueden CT, Schindelin J, Hiner MC, DeZonia BE, Walter AE, Arena ET, Eliceiri KW 2017 ImageJ2: ImageJ for the next generation of scientific image data. BMC Bioinformatics 18:529. doi:10.1186/s12859-017-1934-z. 29187165 PMC5708080

[68] Mülleder M, Capuano F, Pir P, Christen S, Sauer U, Oliver SG, Ralser M 2012 A prototrophic deletion mutant collection for yeast metabolomics and systems biology. Nat Biotechnol 30:1176–1178. doi:10.1038/nbt.2442. 23222782 PMC3520112

[69] Cabrera M, Ungermann C 2008 Purification and in vitro analysis of yeast vacuoles. Methods Enzymol 451:177–196. doi:10.1016/S0076-6879(08)03213-8. 19185721

