# Supplementary material for: A Validated Set of Fluorescent-Protein-Based Markers for Major Organelles in Yeast (Saccharomyces cerevisiae)
Source: mBio. 2019 Sep 3;10(5):e01691-19. doi: 10.1128/mBio.01691-19 (PMC6722415; doi:10.1128/mBio.01691-19)
Supplement: TABLE S1 [file mBio.01691-19-st001.docx]

**Table S1. Fusion constructs with suboptimal properties.**

| Constructs | Description |
| --- | --- |
| **Intended for the endoplasmic reticulum** | |
| DsRedExpress2-HDEL | Weak signal, also present in vacuole lumen. |
| Emc1-mCherry | Localize to the ER. Mediocre signal intensity. |
| Emc1-DuDre | Localize to the ER. Mediocre signal intensity. |
| Sec63-mTagBFP2 | Display a preference for cortical ER. Uneven signal distribution. |
| Slp1-mTagBFP2 | Localize to the plasma membrane. Uneven signal distribution. |
| **Intended for the nucleus** | |
| Nab2-DuDre | Display cytosolic signal in addition to nuclear signal. |
| Opi1-mTagBFP2 | Localize to nuclear envelope and plasma membrane. |
| Opi1(Fragment)-mTagBFP2 | Distribution pattern resembles the ER. |
| Nvj1-mTagBFP2 | Localize to nucleus-vacuole junction and plasma membrane. |
| **Intended for the early Golgi** | |
| Sec21-GFP | Uneven intensity/size distribution among puncta, with some tendency to cluster. |
| Rud3-GFP | Weak signal, few puncta. |
| Rud3-2GFP | Only a few puncta, substantial cytosolic/diffuse signal. |
| Sec21-mCherry | Good signal, but mostly cytosolic/diffuse with inconspicuous puncta. |
| Sec21-DuDre | Multiple puncta with good signal, some of which clearly larger than others. Colocalize with both early and late Golgi green markers. |
| Cop1-mCherry | Good signal, but mostly cytosolic/diffuse with inconspicuous puncta. |
| Cop1-DuDre | Uneven intensity/size distribution among puncta. Colocalize with both early and late Golgi green markers. |
| Vrg4-DuDre | Substantial accumulation in the vacuole lumen. |
| Vrg4-DsRedExpress2 | Primarily in the vacuole lumen. |
| Vrg4-mCherry | Primarily in the vacuole lumen. |
| Rud3-DuDre | Substantial cytosolic signal, weak punctate signal. |
| mCherry-Sed5 | Primarily in the vacuole lumen. |
| Sys1-mCherry | Very weak signal. |
| mCherry-Rer1 | Very weak signal. |
| Cop1-mTagBFP2 | Only a few puncta, with additional plasma membrane distribution. |
| Anp1-mTagBFP2 | Weak punctate signal, with substantial plasma membrane distribution. Expression uneven among cells. |
| Sys1-mTagBFP2 | Localize to the plasma membrane. Expression uneven among cells. |
| Vrg4-mTagBFP2 | Localize to the plasma membrane. |
| Snx4-mTagBFP2 | Localize to the plasma membrane with an uneven distribution. |
| Rud3-mTagBFP2 | No punctate signal. Localize to the plasma membrane, preferentially in the bud. |
| **Intended for late Golgi/early endosomes** | |
| Vps54-GFP | Weak signal, only a few puncta. |
| Mon2-GFP | Weak signal, multiple puncta. |
| Vps54-DuDre | A few faint puncta. |
| Mon2-DuDre | Weak signal, aggregate into large puncta. |
| p1K-mCherry-Tlg1 | Primarily in the vacuole lumen. |
| p1K-mCherry-Tlg2 | Primarily in the vacuole lumen. |
| mCherry-Tlg1 | Strong vacuolar accumulation in addition to punctate signal. |
| pCUP1-DuDre-Tlg1 | No punctate signal. |
| pATG8-mCherry-Tlg1 | Very weak signal. |
| Tvp15-mTagBFP2 | Multiple puncta with good signal, some plasma membrane distribution. Partially colocalize with green early Golgi markers. |
| Mon2-mTagBFP2 | Localize to the plasma membrane. Expression uneven among cells. |
| Sec21-mTagBFP2 | Multiple puncta with good signal, some plasma membrane distribution, some tendency to form aggregate/large puncta. |
| Chs3-mTagBFP2 | Uneven plasma membrane distribution. |
| mTagBFP2-Tlg2 | Uneven plasm membrane distribution. |
| Vps54-mTagBFP2 | Plasma membrane distribution. |
| **Intended for late endosomes** | |
| Snf7-GFP | Substantial presence on vacuole membrane in addition to perivacuolar puncta. |
| Snf7-2GFP | Good signal, but only a few puncta. Moderate presence on vacuole membrane. |
| Vps4-mCherry | Very few puncta, strong cytosolic signal. |
| Snf7-DuDre | Bright and abnormally large puncta. |
| Vps10-mTagBFP2 | Multiple puncta, good signal. Partial colocalization with green late Golgi/early endosome markers. |
| Pep12-mTagBFP2 | Uneven distribution on plasma membrane. |
| Snf7-mTagBFP2 | Localize to an elongated structure positioned between the nucleus and the vacuole. |
| Vps1-mTagBFP2 | Only a few faint puncta, prominent cytosolic signal. |
| Vps8-mTagBFP2 | Only a few faint puncta, also present on plasma membrane. |
| Vps24-mTagBFP2 | A low number of puncta with good signal, some plasma membrane presence. |
| Vps55-mTagBFP2 | A low number of puncta, some plasma membrane presence. |
| Vps60-mTagBFP2 | Localized to the plasma membrane and vacuole. |
| **Intended for the vacuole** | |
| Fet5-GFP | Weak signal, present on both vacuole membrane and endoplasmic reticulum. |
| Fet5-2GFP | Good signal, present on both vacuole membrane and endoplasmic reticulum. |
| Vph1-DuDre | Good signal, localize to the vacuole membrane, but form concentrated patch. |
| Fet5-DuDre | Weak signal. Accumulated inside the vacuole. |
| mCherry-Pho8 | Weak signal, localize to the plasma membrane. |
| **Intended for mitochondria** | |
| Msp1-GFP | Localize to mitochondria, signal weak. |
| Msp1-2GFP | Localize to both mitochondria and peroxisomes. |
| Msp1-mCherry | Weak signal, accumulate inside vacuole. |
| Ldh1-mTagBFP2 | Localize to the plasma membrane. |
| **Intended for peroxisomes** | |
| Pex1-2GFP | Number of puncta lower than expectation, strong cytosolic presence, mediocre signal. |
| Pex1-DuDre | Low number of puncta, weak signal, some accumulation inside the vacuole. |
| DsredExpress2-SKL | Weak signal, localize to vacuole lumen. |
| Pex1-mTagBFP2 | Localize to the plasma membrane. |
| Pex3-mTagBFP2 | Localize to the vacuole and plasma membrane. |
| **Intended for lipid droplets** | |
| Erg6-GFP | Good signal, localize to the ER in addition to puncta. |
| Pet10-GFP | Multiple puncta, a few of which noticeably larger and brighter. |
| Tgl3-DuDre | Low number of puncta, weak signal. |
| Erg6-DuDre | Good signal, some cells contain larger bright droplets. |
| Pet10-DuDre | Low number of puncta, weak signal. Some cells contain larger droplets. |
| Tgl3-mTagBFP2 | Low number of puncta, also present on plasma membrane. |
| Erg6-mTagBFP2 | Lead to larger than normal droplets. |
