# Supplementary material for: A Validated Set of Fluorescent-Protein-Based Markers for Major Organelles in Yeast (Saccharomyces cerevisiae)
Source: mBio. 2019 Sep 3;10(5):e01691-19. doi: 10.1128/mBio.01691-19 (PMC6722415; doi:10.1128/mBio.01691-19)
Supplement: FIG S1 [file mBio.01691-19-sf001.pdf]

Fig. S1

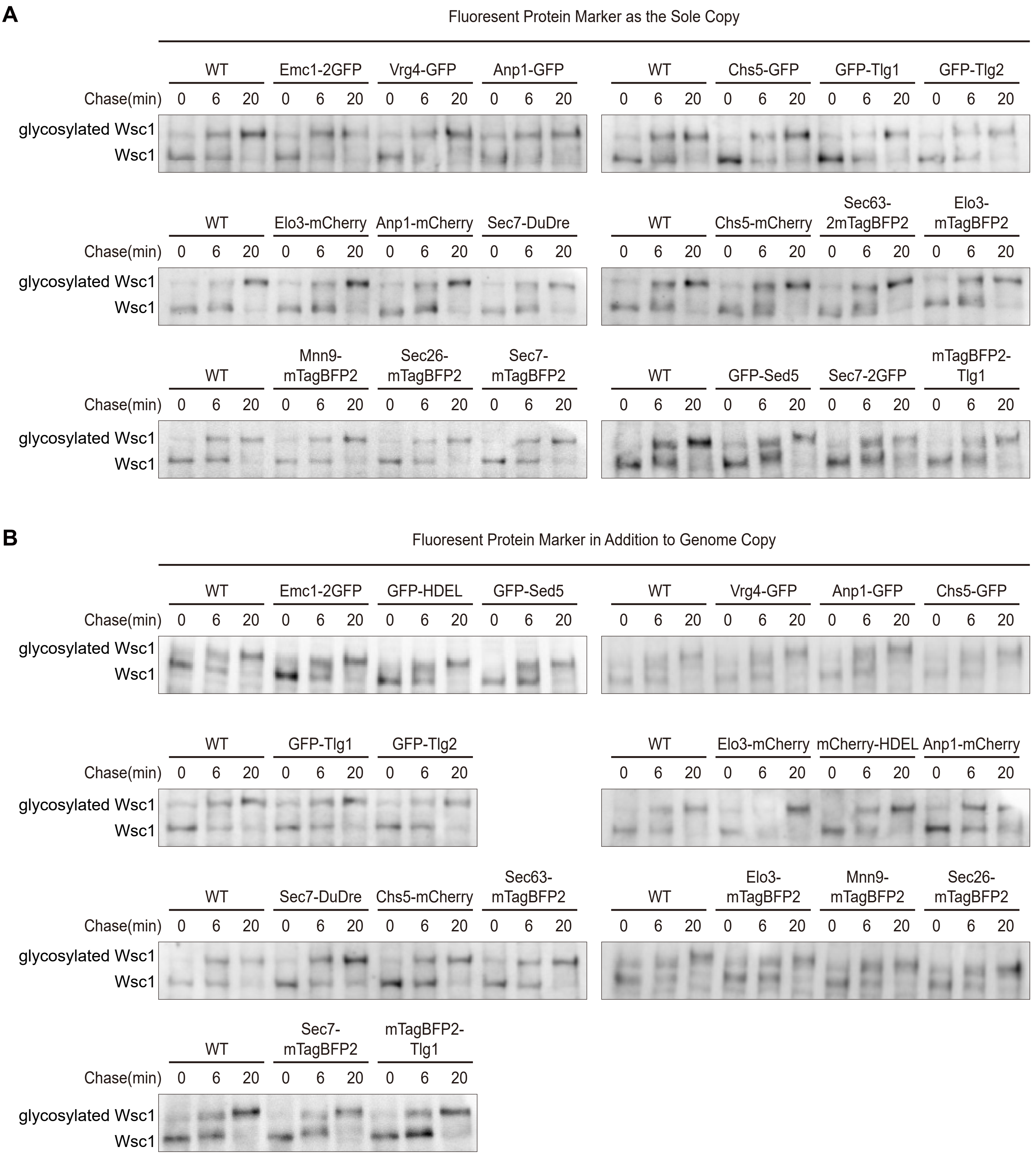
A
Fluoresent Protein Marker as the Sole Copy
WT
Emc1-2GFP
Vrg4-GFP
Anp1-GFP
WT
Chs5-GFP
GFP-Tlg1
GFP-Tlg2
Chase(min)
0 6 20 0 6 20 0 6 20 0 6 20
0 6 20 0 6 20 0 6 20 0 6 20
glycosylated Wsc1
Wsc1
WT
Elo3-mCherry
Anp1-mCherry
Sec7-DuDre
WT
Chs5-mCherry
Sec63-2mTagBFP2
Elo3-mTagBFP2
Chase(min)
0 6 20 0 6 20 0 6 20 0 6 20
0 6 20 0 6 20 0 6 20 0 6 20
glycosylated Wsc1
Wsc1
WT
Mnn9-mTagBFP2
Sec26-mTagBFP2
Sec7-mTagBFP2
WT
GFP-Sed5
Sec7-2GFP
mTagBFP2-Tlg1
Chase(min)
0 6 20 0 6 20 0 6 20 0 6 20
0 6 20 0 6 20 0 6 20 0 6 20
glycosylated Wsc1
Wsc1
B
Fluoresent Protein Marker in Addition to Genome Copy
WT
Emc1-2GFP
GFP-HDEL
GFP-Sed5
WT
Vrg4-GFP
Anp1-GFP
Chs5-GFP
Chase(min)
0 6 20 0 6 20 0 6 20 0 6 20
0 6 20 0 6 20 0 6 20 0 6 20
glycosylated Wsc1
Wsc1
WT
GFP-Tlg1
GFP-Tlg2
WT
Elo3-mCherry
mCherry-HDEL
Anp1-mCherry
Chase(min)
0 6 20 0 6 20 0 6 20
0 6 20 0 6 20 0 6 20 0 6 20
glycosylated Wsc1
Wsc1
WT
Sec7-DuDre
Chs5-mCherry
Sec63-mTagBFP2
WT
Elo3-mTagBFP2
Mnn9-mTagBFP2
Sec26-mTagBFP2
Chase(min)
0 6 20 0 6 20 0 6 20 0 6 20
0 6 20 0 6 20 0 6 20 0 6 20
glycosylated Wsc1
Wsc1
WT
Sec7-mTagBFP2
mTagBFP2-Tlg1
Chase(min)
0 6 20 0 6 20 0 6 20
glycosylated Wsc1
Wsc1
