# Supplementary figures and images for: A Validated Set of Fluorescent-Protein-Based Markers for Major Organelles in Yeast (Saccharomyces cerevisiae)
Source: mBio. 2019 Sep 3;10(5):e01691-19. doi: 10.1128/mBio.01691-19 (PMC6722415; doi:10.1128/mBio.01691-19)

**A**

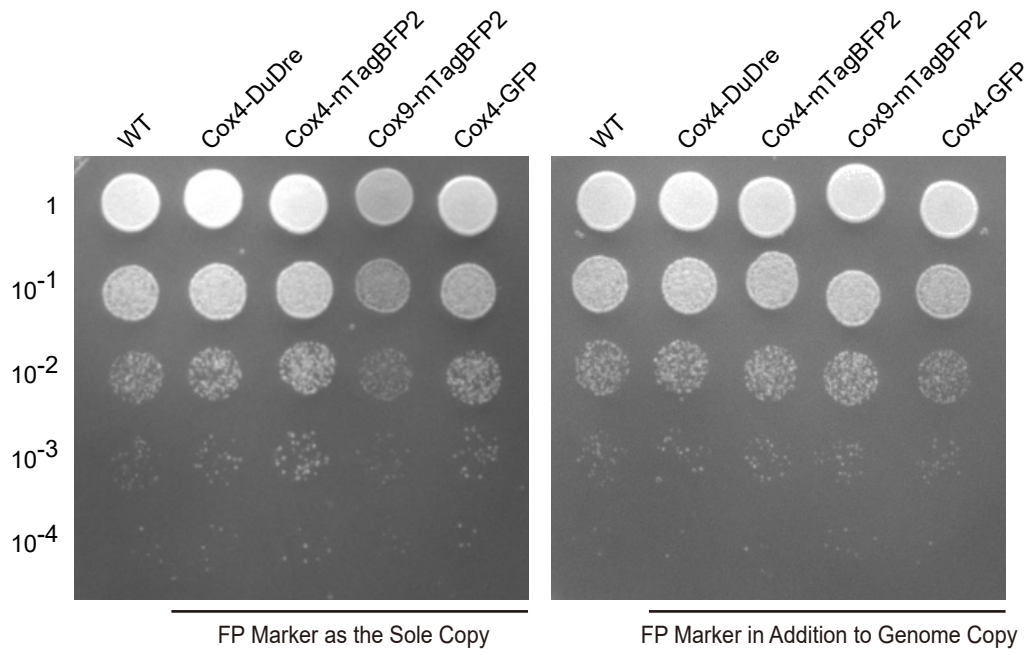

**B**

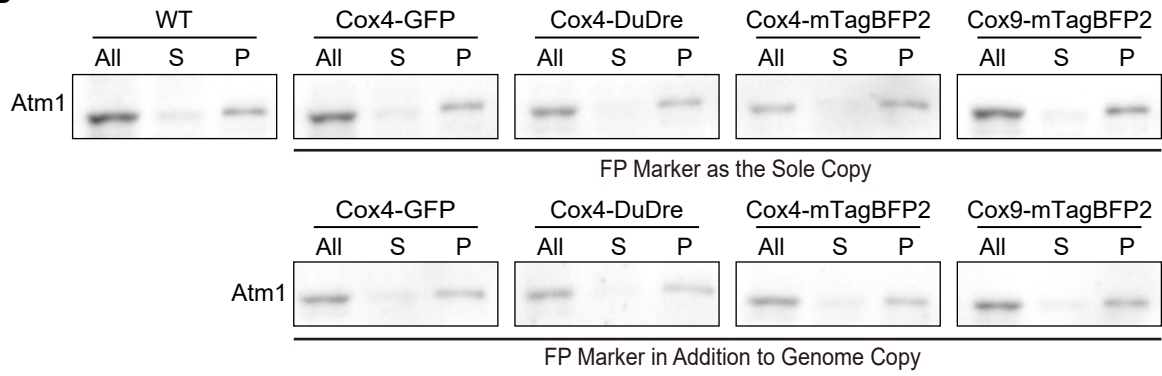

**C**

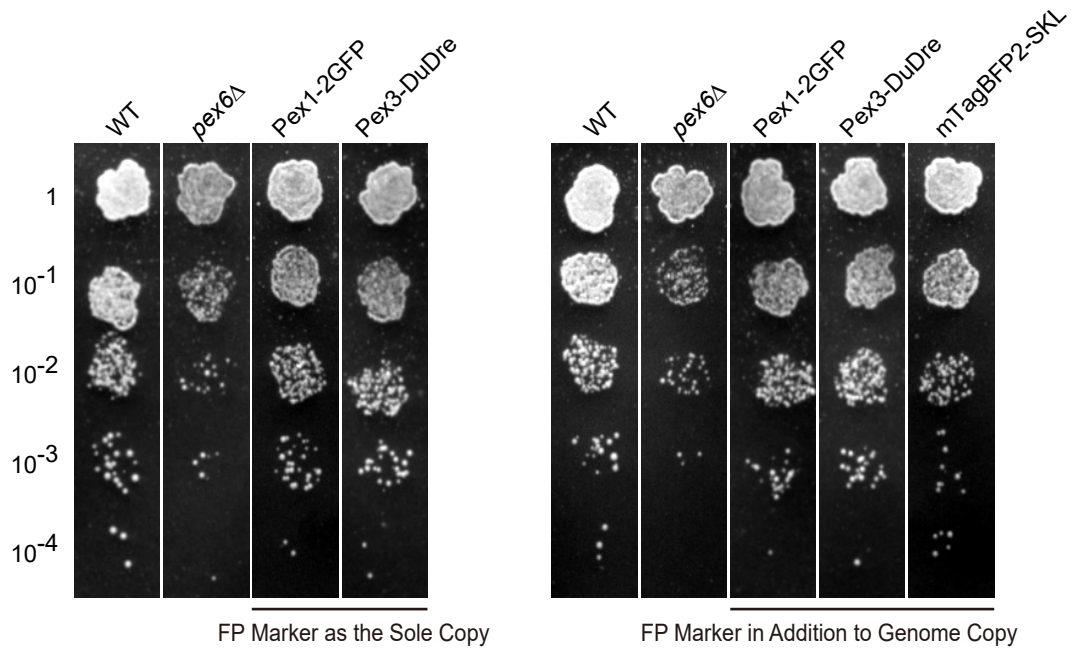

Supplement: FIG S3 [file mBio.01691-19-sf003.pdf]

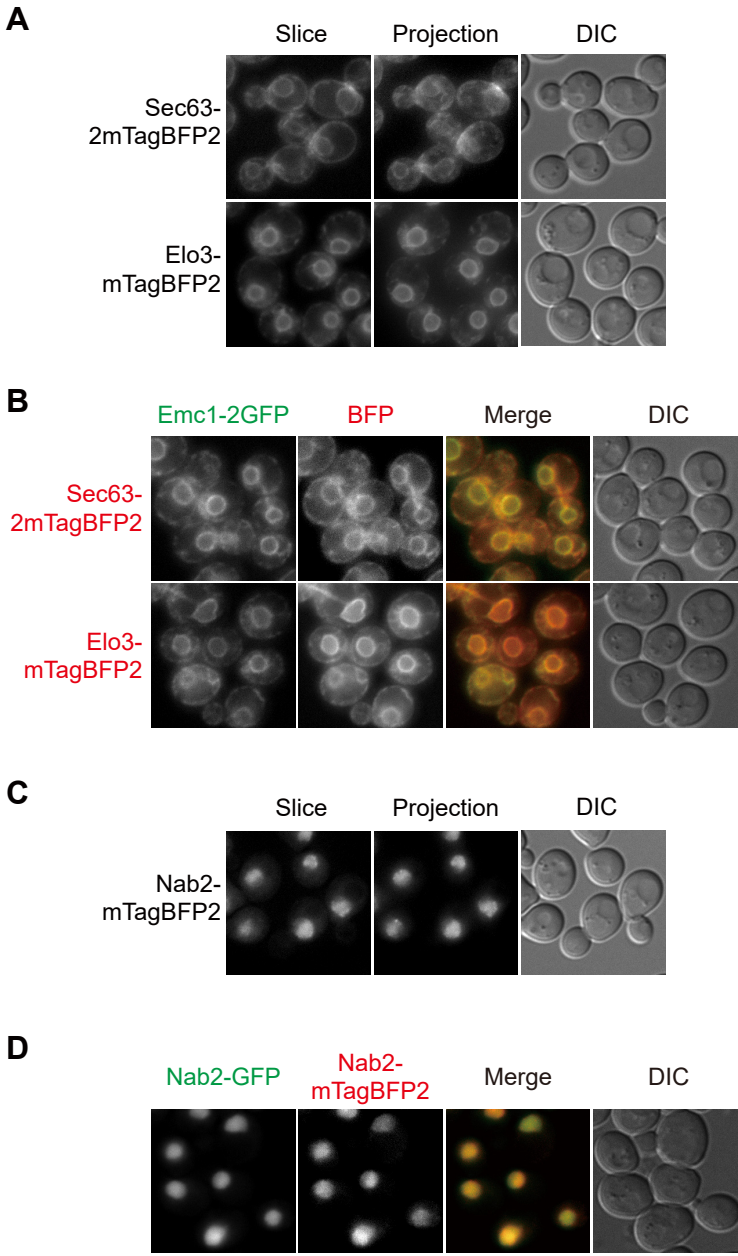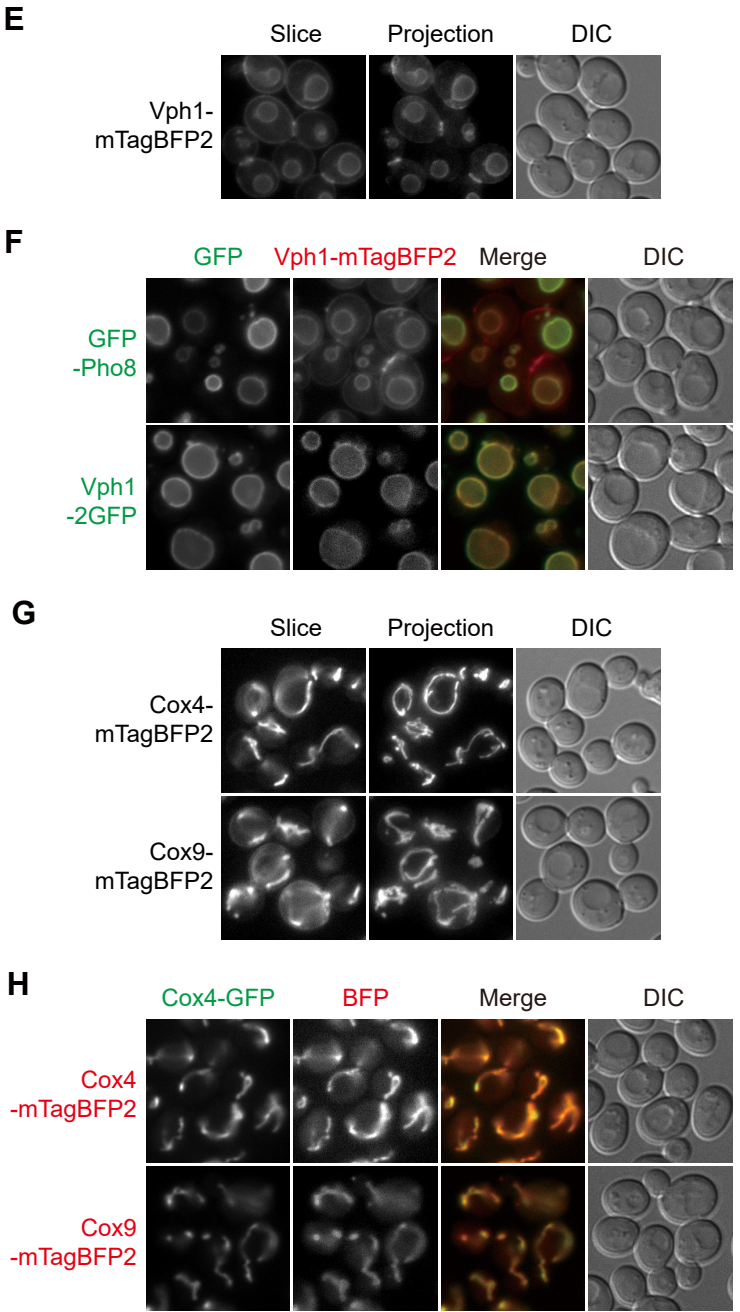

Supplement: FIG S4 [file mBio.01691-19-sf004.pdf]

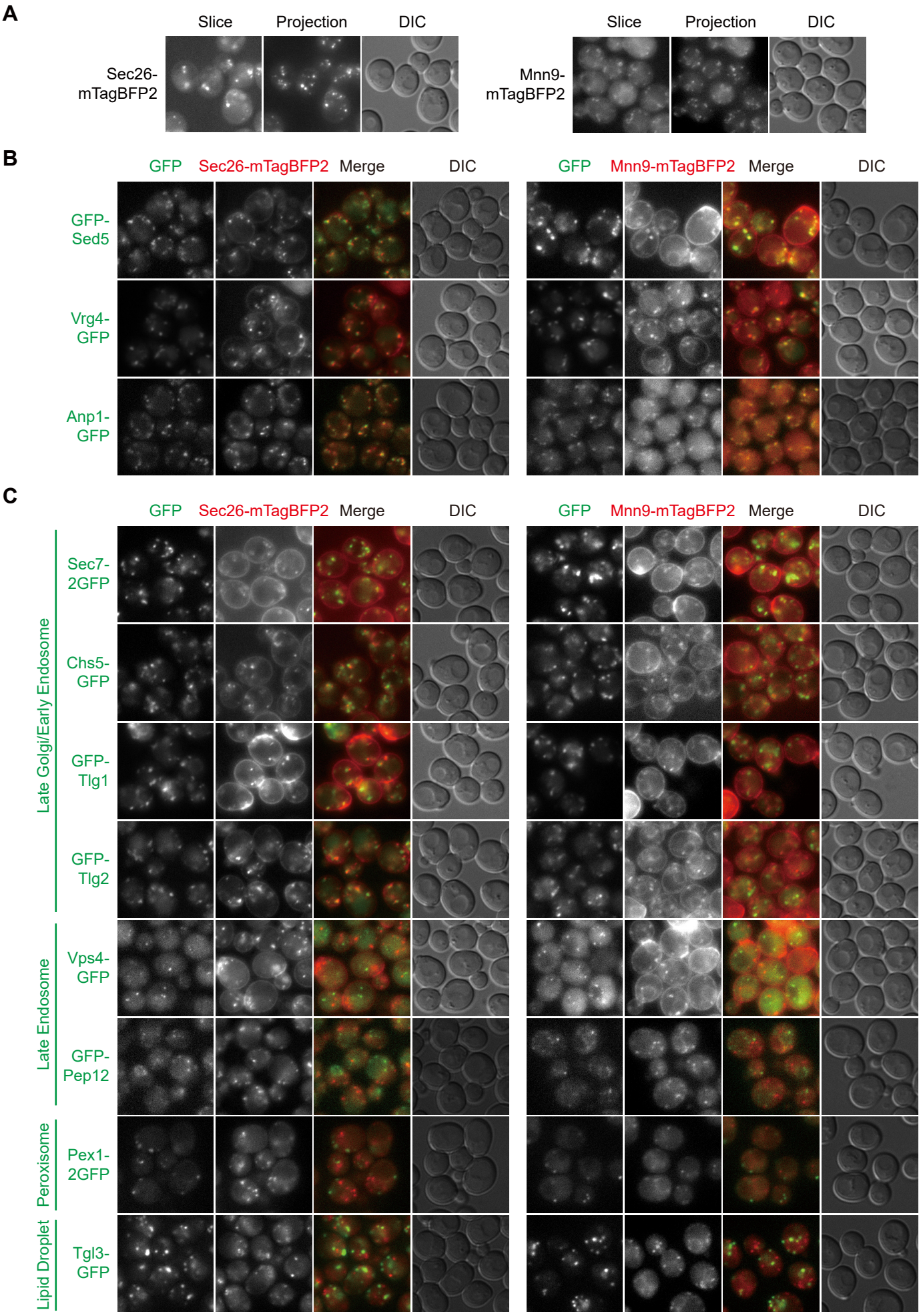

Supplement: FIG S5 [file mBio.01691-19-sf005.pdf]

**A**

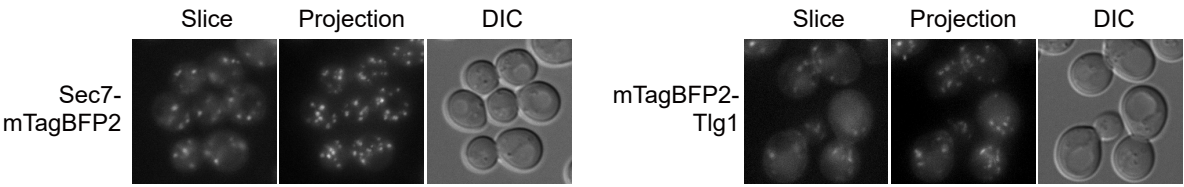

**B**

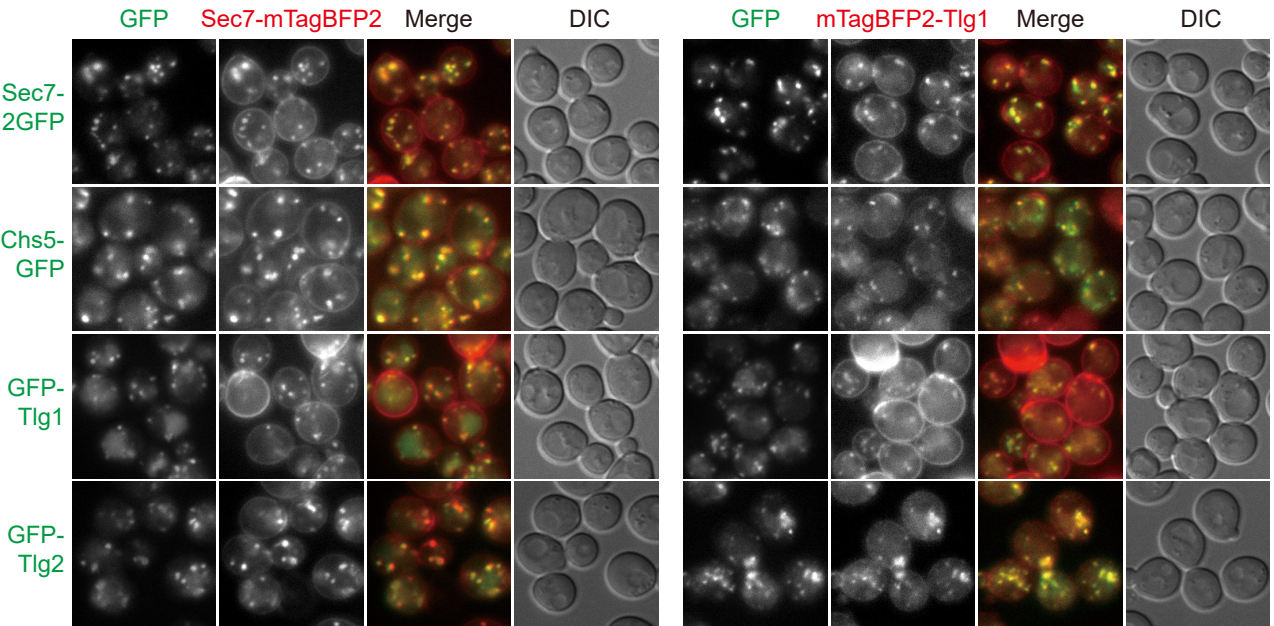

**C**

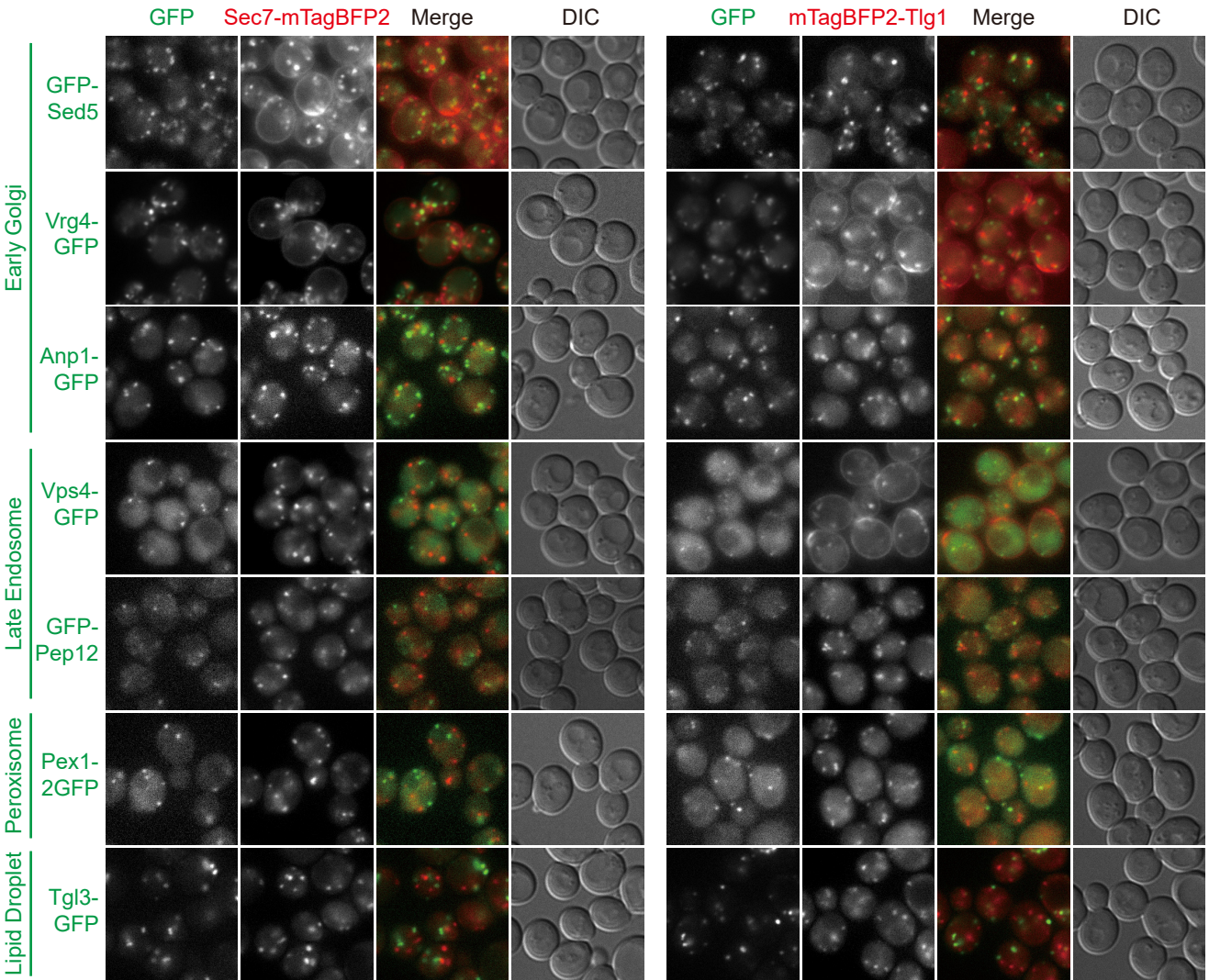

Supplement: FIG S6 [file mBio.01691-19-sf006.pdf]

**Fig. S7**

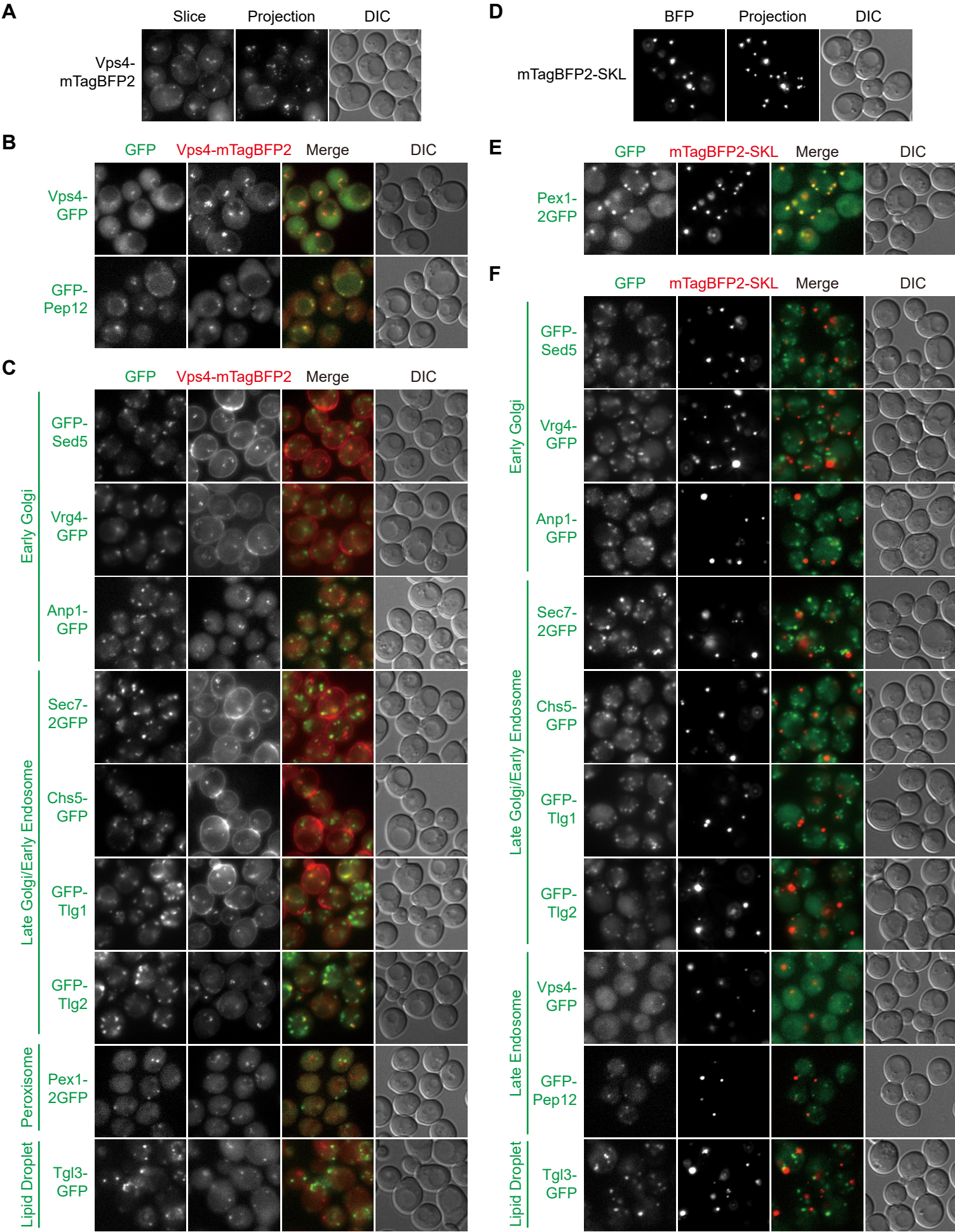

Supplement: FIG S7 [file mBio.01691-19-sf007.pdf]

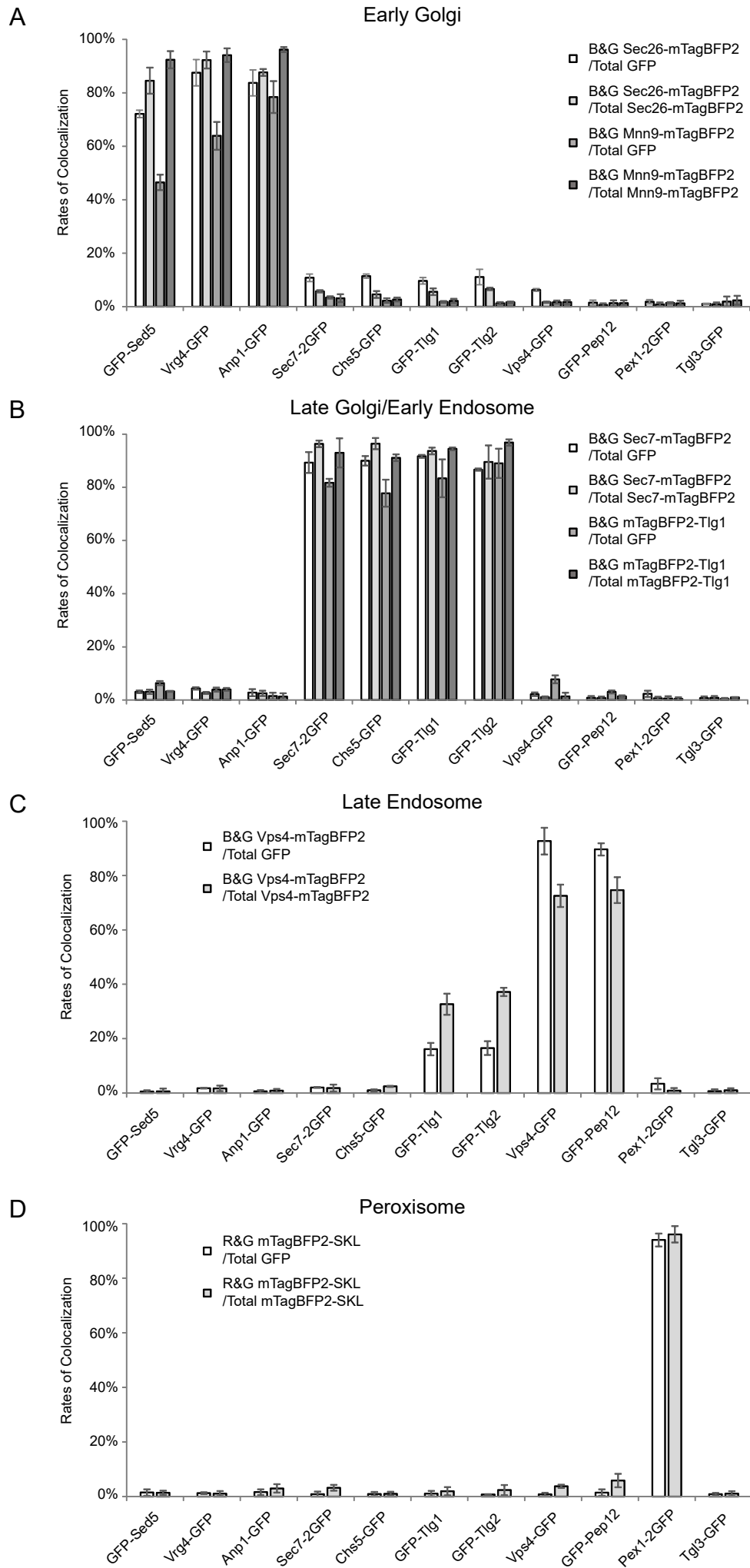

Supplement: FIG S8 [file mBio.01691-19-sf008.pdf]
