# Supplementary material for: A Validated Set of Fluorescent-Protein-Based Markers for Major Organelles in Yeast (Saccharomyces cerevisiae)
Source: mBio. 2019 Sep 3;10(5):e01691-19. doi: 10.1128/mBio.01691-19 (PMC6722415; doi:10.1128/mBio.01691-19)
Supplement: TABLE S2 [file mBio.01691-19-st002.docx]

**Table S2. Construction of plasmids and strains.**

**(A)** Brief descriptions of plasmid construction.

| Plasmid  (selection condition) | Parental plasmid | Restriction sites | Primers for insert amplification |
| --- | --- | --- | --- |
| Emc1-2GFP (Ura) | KT209-2GFP | HindIII | Emc1-GFPF/Emc1-GFPR |
| pTPI1-GFP-HDEL (Ura) | RS406 | SacI & XhoI | pTPI-KCF/pTPI-KCR, yeGFPF/yeGFPR, tAtg8F/tAtg8R |
| Elo3-mCherry (Trp) | Clhn-mCherry-Trp | HindIII | Elo3-mCherryF/Elo3-mCherryR |
| pTPI1-mCherry-HDEL (Trp) | YIPlac204TKC-E2-Crimson-HDEL | NcoI & XbaI | mCherry-HDELF/mCherry-HDELR |
| Sec63-2mTagBFP2 (Trp) | Clhn-2mTagBFP2-Trp | NheI | Sec63-mTagBFP2F/Sec63-mTagBFP2R |
| Elo3-mTagBFP2 (Trp) | Clhn-mTagBFP2-Trp | NheI | Elo3-mTagBFP2F/Elo3-mTagBFP2R |
| Nab2-GFP (Ura) | KT209 | HindIII | Nab2-GFPF/Nab2-GFPR |
| Nab2-mCherry (Trp) | Clhn-mCherry-Trp | HindIII | Nab2-mCherryF/Nab2-mCherryR |
| Nab2-mTagBFP2 (Trp) | Clhn-mTagBFP2-Trp | NheI | Nab2-mTagBFP2F/Nab2-mTagBFP2R |
| GFP-Sed5 (Ura) | Bs-Ura | EcoRI & NotI | pSed5F/pSed5R, GFPF/GFPR, Sed5ORFF/Sed5ORFR |
| Vrg4-GFP (Ura) | KT209 | HindIII | Vrg4-GFPF/Vrg4-GFPR |
| Anp1-GFP (Ura) | KT209 | HindIII | Anp1-GFPF/Anp1-GFPR |
| Anp1-mCherry (Trp) | Clhn-mCherry-Trp | NheI | Anp1-mCherryF/Anp1-mCherryR |
| Mnn9-mTagBFP2 (Trp) | Clhn-mTagBFP2-Trp | NheI | Mnn9-mTagBFP2F/Mnn9-mTagBFP2R |
| Sec26-mTagBFP2 (Trp) | Clhn-mTagBFP2-Trp | NheI | Sec26-mTagBFP2F/Sec26-mTagBFP2R |
| Chs5-GFP (Ura) | KT209 | HindIII | Chs5-GFPF/Chs5-GFPR |
| Sec7-2GFP (Ura) | KT209-2GFP | HindIII & SacII | Sec7-GFPF/Sec7-GFPR, tSec7F/tSec7R |
| pCUP1-GFP-Tlg1 (Ura) | RS406 | SacI & XhoI | pcu-GFPF/pcu-GFPR, GFP-Tlg1F/GFP-Tlg1R |
| pCUP1-GFP-Tlg2 (Ura) | RS406 | SacI & XhoI | pcu-GFPF/pcu-GFPR, GFP-Tlg2F/GFP-Tlg2R |
| Sec7-DuDre (Trp) | Clhn-DuDre-Trp | PvuII | Sec7-DuDreF/Sec7-DuDreR |
| Chs5-mCherry (Trp) | Clhn-mCherry-Trp | HindIII | Chs5-mCherryF/Chs5-mCherryR |
| Sec7-mTagBFP2 (Trp) | Clhn-mTagBFP2-Trp | NheI | Sec7-mTagBFP2F/Sec7-mTagBFP2R |
| mTagBFP2-Tlg1 (Trp) | Clhn-mTagBFP2-Trp | NheI & AscI | pTlg1F/pTlg1R, mTagBFP2F/mTagBFP2R, mTagBFP2-Tlg1F/mTagBFP2-Tlg1R |
| Vps4-GFP (Ura) | KT209 | HindIII | Vps4-GFPF/Vps4-GFPR |
| GFP-Pep12 (Ura) | Bs-Ura | EcoRI & NotI | pPep12F/pPep12R, GFPF/GFPR, Pep12ORFF/Pep12ORFR |
| Vps4-DuDre (Trp) | Clhn-DuDre-Trp | HindIII | Vps4-DuDreF/Vps4-DuDreR |
| Snf7-mCherry (Trp) | Clhn-mCherry-Trp | HindIII | Snf7-mCherryF/Snf7-mCherryR |
| Vps4-mTagBFP2 (Trp) | Clhn-mTagBFP2-Trp | NheI | Vps4-mTagBFP2F/Vps4-mTagBFP2R |
| pCUP1-GFP-Pho8 (Ura) | RS406 | SacI & KpnI | pcu-GFPF/pcu-GFPR, GFP-Pho8F/GFP-Pho8R |
| Vph1-2GFP (Ura) | KT209-2GFP | HindIII & SfiI | Vph1ORF-GFPF/Vph1ORF-GFPR, tVph1F/tVph1R |
| Vph1-mCherry (Trp) | Clhn-mCherry-Trp | HindIII | Vph1-mCherryF/Vph1-mCherryR |
| Vph1-mTagBFP2 (Trp) | Clhn-mTagBFP2-Trp | NheI | Vph1-mTagBFP2F/Vph1-mTagBFP2R |
| Cox4-GFP (Ura) | KT209 | PvuII | Cox4-GFPF/Cox4-GFPR |
| Cox4-DuDre (Trp) | Clhn-DuDre-Trp | PvuII & BsiWI | Cox4-DuDreF/Cox4-DuDreR |
| Cox4-mTagBFP2 (Trp) | Clhn-mTagBFP2-Trp | NheI | Cox4-mTagBFP2F/Cox4-mTagBFP2R |
| Cox9-mTagBFP2 (Trp) | Clhn-mTagBFP2-Trp | NheI | Cox9-mTagBFP2F/Cox9-mTagBFP2R |
| Pex1-2GFP (Ura) | KT209-2GFP | HindIII | Pex1-GFPF/Pex1-GFPR |
| Pex3-DuDre (Trp) | Clhn-DuDre-Trp | HindIII | Pex3-DuDreF/Pex3-DuDreR |
| pTPI1-mTagBFP2-SKL (Trp) | RS404 | KpnI & SacII | pTPI1F/pTPI1R, mTagBFP2F/mTagBFP2R, tTPI1F/tTPI1R |
| Tgl3-GFP (Ura) | KT209 | HindIII | Tgl3-GFPF/Tgl3-GFPR |
| Tgl3-mCherry (Trp) | Clhn-mCherry-Trp | HindIII | Tgl3-mCherryF/Tgl3-mCherryR |
| Erg6-mCherry (Trp) | Clhn-mCherry-Trp | HindIII | Erg6-mCherryF/Erg6-mCherryR |

**(B)** DNA sequences of PCR primers.

| **Primer name** | **Sequence** |
| --- | --- |
| BS-UraF | CCTTTTTTGCGAGGCATATTTATG |
| BS-UraR | GTAACTATTGAATTTTGTTTGGATTT |
| pSed5F | GATAAGCTTGATATCGAATTCCTATCGCCTCAAAGAACCACAT |
| pSed5R | AATTCTTCACCTTTAGACATGGGAGTTGTGTGGTATGGTGA |
| GFPF | ATGTCTAAAGGTGAAGAATTATTCA |
| GFPR | GTTAATTAACCCGGGGATCCGTC |
| Sed5ORFF | GGATCCCCGGGTTAATTAACATGAACATAAAGGATAGAACTTCAG |
| Sed5ORFR | AATACAGTTTTTTGCGGCCGCTTAATTGACTAAAACCCAAATAACG |
| Vrg4-GFPF | CGCGGCCGCCAGCTGAAGCTTGATCTCAAGGAAAAAAATGCAATG |
| Vrg4-GFPR | CGACCTGCAGCGTACGAAGCTTTACGTAAAGGTTGGGCTTGTT |
| Anp1-GFPF | GAACGCGGCCGCCAGCTGAAGATTCCAATGTCAAGATATGGCA |
| Anp1-GFPR | AATTCTTCACCTTTAGACATTTCTTCAGCAACTTCAAGAGAAAGGTTTCTATCAGGGTCGAAGTCT |
| Anp1-mCherryF | GGGTTAATTAACATCGCTAGCGATTCCAATGTCAAGATATGGCA |
| Anp1-mCherryR | TCCTCGCCCTTGCTCACCATTTCTTCAGCAACTTCAAGAGAAAGGTTTCTATCAGGGTCGAAGTCT |
| Mnn9-mTagBFP2F | GGGTTAATTAACATCGCTAGCAATTCTAGCGTAGGATCAAGTGC |
| Mnn9-mTagBFP2R | TCTTTGATCAGTTCGCTCATTTCTTCAGCAACTTCAAGAGAAAGATGGTTCTCTTCCTCTATGTGAT |
| Sec26-mTagBFP2F | GGTTAATTAACATCGCTAGCTTCACCGTTAACATCTGTCGTAT |
| Sec26-mTagBFP2R | TCTTTGATCAGTTCGCTCATTTCTTCAGCAACTTCAAGAGAAAGAACATGAGTGAGAGCAAGTTTATT |
| Cop1-mTagBFP2F | GGTTAATTAACATCGCTAGCCTGCCAACGACTTCGTCAGG |
| Cop1-mTagBFP2R | TCTTTGATCAGTTCGCTCATTTCTTCAGCAACTTCAAGAGAAAGTACACGTATTCTTAATCCGGATG |
| Chs5-GFPF | CGCGGCCGCCAGCTGAAGCTTCTAACTATAATAACTGGTAGCTTTG |
| Chs5-GFPR | AATTCTTCACCTTTAGACATGTTAATTAAACCAGCACCGTCAC |
| Sec7-GFPF | TAGAACGCGGCCGCCAGCTGAAGCTTGATATTGCAAAACGTATCTAAATTC |
| Sec7-GFPR | CCGTCGACCTGCAGCGTACGAAGCATCAGTAGAAAGGTATAATTCACC |
| tSec7F | TAGTGGCCTATGCGGCCGCGGCAGATTAAAATATGCTTAGTTGTAG |
| tSec7R | CTATAGGGAGACCGGCAGATCCGCGGCCAAAAGTTGCCTGTCCAAAGA |
| Sec7-DuDreF | ATAGAACGCGGCCGCCAGCTGAAAATGTTGAGATAATTGTTGGGATTC |
| Sec7-DuDreR | CGACCTGCAGCGTACGAAGCATCAGTAGAAAGGTATAATTCACC |
| Chs5-mCherryF | CGCGGCCGCCAGCTGAAGCTTCTAACTATAATAACTGGTAGCTTTG |
| Chs5-mCherryR | GACCTGCAGCGTACGaagCTTTCTTTTTCCCTTTCTTCTTATTCTT |
| Sec7-mTagBFP2F | GGGTTAATTAACATCGCTAGCAATGTTGAGATAATTGTTGGGATTC |
| Sec7-mTagBFP2R | TCTTTGATCAGTTCGCTCATTTCTTCAGCAACTTCAAGAGAAAGATCAGTAGAAAGGTATAATTCACC |
| pcu-GFPF | AGGGAACAAAAGCTGGAGCTCCTAGTTAGAAAAAGACATTTTTGCT |
| pcu-GFPR | ATGTCACTTGTTCCGGCTCCTTTGTACAATTCATCCATACCATG |
| GFP-Tlg1F | GGAGCCGGAACAAGTGACATGAAGATGAACAACAGTGAAGATCCGTT |
| GFP-Tlg1R | GGTACCGGGCCCCCCCTCGAGCTAAGCAATGAATGCCAAAACTAA |
| GFP-Tlg2F | GGAGCCGGAACAAGTGACATGAAGATGTTTAGAGATAGAACTAATTTATTT |
| GFP-Tlg2R | GGTACCGGGCCCCCCCTCGAGCTAAAGTAGGTCATCCAAAGCAT |
| pTlg1F | GGGTTAATTAACATCGCTAGCGATAGTCCCCCATTTTTTTTTATG |
| pTlg1R | TCTTTGATCAGTTCGCTCATTTGTTAAAGAAAGGATCTTAGCAAT |
| mTagBFP2F | ATGAGCGAACTGATCAAAGAGAA |
| mTagBFP2R | TTCAGCAACTTCAAGAGAAAGATTCAGTTTATGACCCAGCTTG |
| mTagBFP2-Tlg1F | TTTCTCTTGAAGTTGCTGAAGAAATGAACAACAGTGAAGATCCGTT |
| mTagBFP2-Tlg1R | TTTAGAAGTGGCGCGCCTCAAGCAATGAATGCCAAAACTAATAAA |
| Vps4-GFPF | TAGAACGCGGCCGCCAGCTGAAGCTTCATCACTTTTGTTCCTTTTGTCG |
| Vps4-GFPR | AATTCTTCACCTTTAGACATGTTAATTAAACCAGCACCGTCAC |
| pPep12F | TTCCTGCAGCCCGGGGGATCCTACTTAGTCCTTTGGTCAAAAATAT |
| pPep12R | AATTCTTCACCTTTAGACATCTCAACACAATTATTGTAGTAATTTA |
| Pep12ORFF | GGATCCCCGGGTTAATTAACATGTCGGAAGACGAATTTTTTGG |
| Pep12ORFR | AATACAGTTTTTTGCGGCCGCTTACAATTTCATAATGAGAAAAATAAAA |
| Vps4-DuDreF | CGCGGCCGCCAGCTGAAgcttCATCACTTTTGTTCCTTTTGTCG |
| Vps4-DuDreR | TCCCACGACCTGCAGCGTACGAAGCGTTACCTTCTTGACCAA |
| Snf7-mCherryF | CGCGGCCGCCAGCTGAAGCTTATGCGTTGATTATTGGGTTTCTC |
| Snf7-mCherryR | CCCACGACCTGCAGCGTACGAAGCAAGCCCCATTTCTGCTTGTAGT |
| Vps4-mTagBFP2F | GGGTTAATTAACATCGCTAGCCATCACTTTTGTTCCTTTTGTCG |
| Vps4-mTagBFP2R | TCTTTGATCAGTTCGCTCATTTCTTCAGCAACTTCAAGAGAAAGGTTACCTTCTTGACCAAAATCTC |
| Pex1-GFPF | CGCGGCCGCCAGCTGAAGCTTATGACAGCAGCAAGGGAAACAA |
| Pex1-GFPR | CCGTCGACCTGCAGCGTACGAAGCCATAAGGGAGAGTCGGCTACC |
| Pex3-DuDreF | CGCGGCCGCCAGCTGAAGCTTTGTTAATCATTATCCATTTTTGCTTT |
| Pex3-DuDreR | CCCACGACCTGCAGCGTACGaagcAGGCTTGAAGGAAAACGAGCTG |
| Tgl3-GFPF | CGCGGCCGCCAGCTGAAGCTTGACTGGCTGATAGCCTCGAC |
| Tgl3-GFPR | CCGTCGACCTGCAGCGTACGAAGCCCTACTCCGTCTTGCTCTTATT |
| pTPI1F | TACAAAAAACACATACATAAACTAAAAATGAGCGAACTGATCAAAGA |
| pTPI1R | CTTTGATCAGTTCGCTCATGTCGACAAAGAAAGTTCTAGCCATTTTTAGTTTATGTATGTGTTTTTTGTA |
| tTPI1F | AGCTGGGTCATAAACTGAATTCCAAACTATAGAGATAATATTTTTATATAATTATATTAATC |
| tTPI1R | AAGCTGGAGCTCCACCGCGGGTGGGATTTGAATAGAAACGACG |
| Tgl3-mCherryF | CGCGGCCGCCAGCTGAAGCTTGACTGGCTGATAGCCTCGAC |
| Tgl3-mCherryR | CCACGACCTGCAGCGTACGaagCCCTACTCCGTCTTGCTCTTATT |
| Erg6-mCherryF | GAACGCGGCCGCCAGCTGAATGCTCGCTATCCTCGCCATC |
| Erg6-mCherryR | CGACCTGCAGCGTACGAAGCTTGAGTTGCTTCTTGGGAAGTTT |
| Emc1-GFPF | ACGCGGCCGCCAGCTGAAGCTTGCTACTTTCTCAGTCACTGTT |
| Emc1-GFPR | CGACCTGCAGCGTACGAAGCTTTAATTAGCCATTGGGATTTCAAC |
| Emc1-mCherryF | GGGTTAATTAACATCGCTAGCTGGGGGTTGTTTGGCTCTTTG |
| Emc1-mCherryR | TCCTCGCCCTTGCTCACCATTTCTTCAGCAACTTCAAGAGAAAGTTTAATTAGCCATTGGGATTTCAAC |
| Emc1-DuDreF | CGCGGCCGCCAGCTGAAgcttTGGGGGTTGTTTGGCTCTTTGC |
| Emc1-DuDreR | ACGACCTGCAGCGTACGAAGCTTTAATTAGCCATTGGGATTTCAAC |
| Sec63-mTagBFP2F | GGGTTAATTAACATCGCTAGCGTTTTGACGCGATGTTTCCTCT |
| Sec63-mTagBFP2R | TCTTTGATCAGTTCGCTCATTTCTTCAGCAACTTCAAGAGAAAGTTCTGGTGATTCATCATCTTCAG |
| Elo3-mTagBFP2F | GGGTTAATTAACATCGCTAGCGTAAAAAGGGTTTTCAGATCGTTA |
| Elo3-mTagBFP2R | TCTTTGATCAGTTCGCTCATTTCTTCAGCAACTTCAAGAGAAAGAGCTTTCCTGGAAGAGACCTTG |
| Nab2-GFPF | CGCGGCCGCCAGCTGAAGCTTACTAAAATCATAGCGCCCTGTAT |
| Nab2-GFPR | CGACCTGCAGCGTACGAAGCGTTCATTTCCGTATCTTGTTCTTG |
| Nab2-mCherryF | GGGTTAATTAACATCGCTAGCACTAAAATCATAGCGCCCTGTAT |
| Nab2-mCherryR | TCCTCGCCCTTGCTCACCATTTCTTCAGCAACTTCAAGAGAAAGGTTCATTTCCGTATCTTGTTCTTG |
| Nab2-mTagBFP2F | GGGTTAATTAACATCGCTAGCACTAAAATCATAGCGCCCTGTAT |
| Nab2-mTagBFP2R | TCTTTGATCAGTTCGCTCATTTCTTCAGCAACTTCAAGAGAAAGGTTCATTTCCGTATCTTGTTCTTG |
| Cox4-GFPF | AGAACGCGGCCGCCAGCTGAAGCCTATTGGATTGGCCCAATTG |
| Cox4-GFPR | AATTCTTCACCTTTAGACATTTCTTCAGCAACTTCAAGAGAAAGGTGATGGTGGTCATCATTTGGA |
| Cox4-DuDreF | ATAGAACGCGGCCGCCAGCTGAAGCCTATTGGATTGGCCCAATTG |
| Cox4-DuDreR | ACGACCTGCAGCGTACGAAGCGTGATGGTGGTCATCATTTGGA |
| Cox4-mTagBFP2F | GGGTTAATTAACATCGCTAGCGCCTATTGGATTGGCCCAATTG |
| Cox4-mTagBFP2R | TCTTTGATCAGTTCGCTCATTTCTTCAGCAACTTCAAGAGAAAGGTGATGGTGGTCATCATTTGGA |
| Cox9-mTagBFP2F | GGGTTAATTAACATCGCTAGCACACTTTCAAAGTTTGTGGTTGAG |
| Cox9-mTagBFP2R | TCTTTGATCAGTTCGCTCATTTCTTCAGCAACTTCAAGAGAAAGGTTCTCTTGCTTTTTCCTCTCAG |
| GFP-Pho8F | AAGGAGCCGGAACAAGTGACATGATGACTCACACATTACCAAG |
| GFP-Pho8R | CTATAGGGCGAATTGGGTACCGCGCTTCACTTAACGACGATG |
| Vph1ORF-GFPF | CGCGGCCGCCAGCTGAAGCTTGCTAGCTTTTACCTTTCTCTATACA |
| Vph1ORF-GFPR | CGACCTGCAGCGTACGAAGCGCTTGAAGCGGAAGAGCTTGC |
| tVph1F | ATATCGATGATATCAGATCCACTAGTGAGGACTTTTAAAAAAAGC |
| tVph1R | AGGGAGACCGGCAGATCCGCGGCCGCATAGGCCACTAGTAGGCCTCATTCCCAAT |
| Vph1-mCherryF | CGCGGCCGCCAGCTGAAGCTTTGTGGTGTTGCGTTAGGTCTAA |
| Vph1-mCherryR | CCCACGACCTGCAGCGTACGAAGCGCTTGAAGCGGAAGAGCTTGC |
| Vph1-mTagBFP2F | GGGTTAATTAACATCGCTAGCTGTGGTGTTGCGTTAGGTCTAA |
| Vph1-mTagBFP2R | TCTTTGATCAGTTCGCTCATTTCTTCAGCAACTTCAAGAGAAAGGCTTGAAGCGGAAGAGCTTGC |
| Emc1-2GFP recombination F | GGGTAAATTGCTTATTACTATATTC |
| Emc1-2GFP recombination R | AATGTGTATCTGATATATATAATGTGTATGTAAATATCTATAATACGACTCACTATAGG |
| Nab2-GFP/mcherry/BFP recombination F | TACCAATGAAAGGCCATTTGCAT |
| Nab2-GFP/mcherry/BFP recombination R | CAGGAACATGAATTTCGTTCCGTGATTTTAATAGTAATCATAATACGACTCACTATAGG |
| Vrg4-GFP recombination F | AAACTTCTTATCTATTCTCTCCATT |
| Vrg4-GFP recombination R | CCAAAAAAAAAAAATAAATTGCACCCCCCGTAAGTTCTCATAATACGACTCACTATAGG |
| Anp1-GFP/mcherry recombination F | GCTCGACGGCAACCCGCAG |
| Anp1-GFP/mcherry recombination R | ATTCGTACTTCATATGTAGGTCACTAAAAAACCGAGCCTATAATACGACTCACTATAGG |
| Chs5-GFP/mcherry recombination F | AGATGACGTTTTGTCCACTAAAG |
| Chs5-GFP/mcherry recombination R | AACGTGCGTCGTGGAACTCATTGAAGGCATCCATTAATCATAATACGACTCACTATAGG |
| GFP-Tlg1 recombination F | AACTGTAAAATTGTATTGCTAAGATCCTTTCTTTAACAACCTGATGCGGTATTTTCTCC |
| GFP-Tlg1 recombination R | CACCAGCAGTATTGTGACGAG |
| GFP-Tlg2 recombination F | ACATTTTATCGCTTTGTGATCTAGGCAGTCGTTACAAACCCTGATGCGGTATTTTCTCC |
| GFP-Tlg2 recombination R | CTTGATCATCGCCCAAGGGTG |
| Vps4-GFP/DuDre/BFP recombination F | GGATTTCTTAAAGGCTATCAAATC |
| Vps4-GFP/DuDre/BFP recombination R | TACACAAGAAATCTACATTAGCACGTTAATCAATTGACTATAATACGACTCACTATAGG |
| GFP-Pho8 recombination F | ATCAGCATACGGGACATTATTTGAACGCGCATTAGCAGCCCTGATGCGGTATTTTCTCC |
| GFP-Pho8 recombination R | TCTTTGATCTCTTCGAGATCCG |
| Cox4-GFP/DuDre/BFP recombination F | GTGGTTAAAACCAACTGTTAACG |
| Cox4-GFP/DuDre/BFP recombination R | GTAAAAGAGAAACAGAAGGGCAACTTGAATGATAAGATTATAATACGACTCACTATAGG |
| Pex1-2GFP recombination F | CAAATTAAGGGGGATTTACGATAG |
| Pex1-2GFP recombination R | TAAAGGGAAACGCGCTTTGTTCTTTTCTTCTTCCTTTTCATAATACGACTCACTATAGG |
| Tgl3-GFP/mcherry recombination F | ATCTGGTGAACGAAGTACATGG |
| Tgl3-GFP/cherry recombination R | AATAAAAAAAATAAGACAGAAAAAAGTGGAAACGATACTATAATACGACTCACTATAGG |
| Elo3-mCherry/BFP recombination F | AAAGACAGTCAAGAAGGAATCTG |
| Elo3-mCherry/BFP recombination R | TCTTTTTCATTCGCTGTCAAAAATTCTCGCTTCCTATTTATAATACGACTCACTATAGG |
| Sec7-DuDre/BFP recombination F | TCTGGTGATTCAAATATTAGATAAG |
| Sec7-DuDre/BFP recombination R | AAGCATATTTTAATCTGCTGGACCATTCAACAAAGCCTTATAATACGACTCACTATAGG |
| Snf7-mCherry recombination F | TAAACAAAGTGAGAACTCTGTGAA |
| Snf7-mCherry recombination R | CTTTTTTTTTCTTTCATCTAAACCGCATAGAACACGTTCATAATACGACTCACTATAGG |
| Vph1-mCherry/BFP recombination F | CAAGTTTTTCGTGGGTGAAGGT |
| Vph1-mCherry/BFP recombination R | ACTTAAATGTTTCGCTTTTTTTAAAAGTCCTCAAAATTTATAATACGACTCACTATAGG |
| Pex3-DuDre recombination F | CGAATACCTGGCCACTCTGG |
| Pex3-DuDre recombination R | ATATATTCTGGTGTGAGTGTCAGTACTTATTCAGAGATTATAATACGACTCACTATAGG |
| Erg6-mCherry recombination F | CGGTGGTAAGTCCAAGTTATTC |
| Erg6-mCherry recombination R | TCGTGCGCTTTATTTGAATCTTATTGATCTAGTGAATTTATAATACGACTCACTATAGG |
| Sec63-2mTagBFP2 recombination F | TACTGATGACGACGAAACCGAA |
| Sec63-2mTagBFP2 recombination R | AAGAGCTAAAATGAAAAACTATACTAATCACTTATATCTATAATACGACTCACTATAGG |
| Mnn9-mTagBFP2 recombination F | GATTGAAACAGAAGGTTTTGCTAA |
| Mnn9-mTagBFP2 recombination R | TAACGCTATAGCTTCTGTATGCTTTTTGCTCAGTTGCTCATAATACGACTCACTATAGG |
| Sec26-mTagBFP2 recombination F | TGTTCGTATCCGATCAAAGGGA |
| Sec26-mTagBFP2 recombination R | TGACATTATAACTTACATTGCATCAGCTCAGGGTGGTTCATAATACGACTCACTATAGG |
| Cox9-mTagBFP2F | TGTCATGGCCTCTTACTGGTG |
| Cox9-mTagBFP2 recombination R | AGAATATAATGCGAAAAACAATAGTGGTCAGGTTCGGTCATAATACGACTCACTATAGG |
