# Supplementary material for: A Validated Set of Fluorescent-Protein-Based Markers for Major Organelles in Yeast (Saccharomyces cerevisiae)
Source: mBio. 2019 Sep 3;10(5):e01691-19. doi: 10.1128/mBio.01691-19 (PMC6722415; doi:10.1128/mBio.01691-19)
Supplement: FIG S2 [file mBio.01691-19-sf002.pdf]

Fig. S2

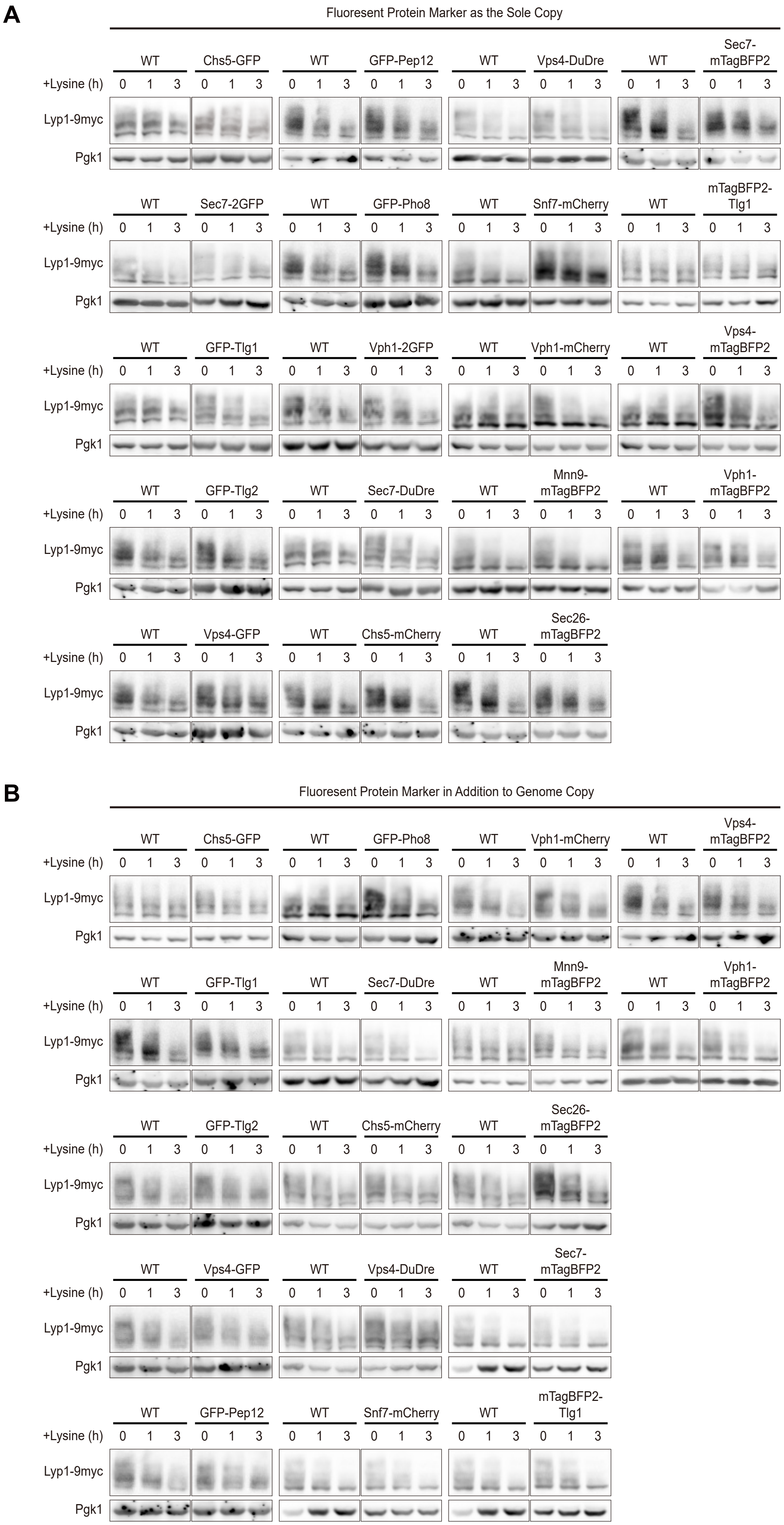
A
Fluoresent Protein Marker as the Sole Copy
WT
Chs5-GFP
GFP-Pep12
Vps4-DuDre
Sec7-mTagBFP2
Sec7-2GFP
GFP-Pho8
Snf7-mCherry
mTagBFP2-Tlg1
GFP-Tlg1
Vph1-2GFP
Vph1-mCherry
Vps4-mTagBFP2
GFP-Tlg2
Sec7-DuDre
Mnn9-mTagBFP2
Vph1-mTagBFP2
Vps4-GFP
Chs5-mCherry
Sec26-mTagBFP2
+Lysine (h)
0 1 3
Lyp1-9myc
Pgk1
B
Fluoresent Protein Marker in Addition to Genome Copy
WT
Chs5-GFP
GFP-Pho8
Vph1-mCherry
Vps4-mTagBFP2
GFP-Tlg1
Sec7-DuDre
Mnn9-mTagBFP2
Vph1-mTagBFP2
GFP-Tlg2
Chs5-mCherry
Sec26-mTagBFP2
Vps4-GFP
Vps4-DuDre
Sec7-mTagBFP2
GFP-Pep12
Snf7-mCherry
mTagBFP2-Tlg1
+Lysine (h)
0 1 3
Lyp1-9myc
Pgk1
